# Targeting histone methyltransferase G9a inhibits growth and Wnt signaling pathway by epigenetically regulating HP1α and APC2 gene expression in non-small cell lung cancer

**DOI:** 10.1186/s12943-018-0896-8

**Published:** 2018-10-22

**Authors:** Keqiang Zhang, Jinhui Wang, Lu Yang, Yate-Ching Yuan, Tommy R. Tong, Jun Wu, Xinwei Yun, Melissa Bonner, Rajendra Pangeni, Zheng Liu, Tiger Yuchi, Jae Y. Kim, Dan J. Raz

**Affiliations:** 1 Division of Thoracic Surgery, City of Hope Medical Center, Duarte, CA USA; 2 The Integrative Genomics Core lab of Department of Molecular Medicine, City of Hope National Medical Center, Duarte, CA USA; 3 The Bioinformatics Core lab of Department of Molecular Medicine, City of Hope Medical Center, Duarte, CA USA; 4 Department of Pathology, City of Hope Medical Center, Duarte, CA USA; 5 Division of Comparative Medicine, City of Hope National Medical Center, Duarte, CA USA

**Keywords:** NSCLC, histone methyltransferase G9a, HP1α, APC2, Cell growth, Wnt signaling pathway

## Abstract

**Background:**

Dysregulated histone methyltransferase G9a may represent a potential cancer therapeutic target. The roles of G9a in tumorigenesis and therapeutics are not well understood in non-small cell lung cancer (NSCLC). Here we investigated the impact of G9a on tumor growth and signaling pathways in NSCLC.

**Methods:**

Immunohistochemistry analyzed G9a expression in NSCLC tissues. Both siRNA and selective inhibitor were used to target G9a. The impact of targeting G9a on key genes, signaling pathways and growth were investigated in NSCLC cells by RNA sequencing analysis, rescue experiments, and xenograft models.

**Results:**

Overexpression of G9a (≥ 5% of cancer cells showing positive staining) was found in 43.2% of 213 NSCLC tissues. Multiple tumor-associated genes including HP1α, APC2 are differentially expressed; and signaling pathways involved in cellular growth, adhesion, angiogenesis, hypoxia, apoptosis, and canonical Wnt signaling pathways are significantly altered in A549, H1299, and H1975 cells upon G9a knockdown*.* Additionally, targeting G9a by siRNA-mediated knockdown or by a selective G9a inhibitor UNC0638 significantly inhibited tumor growth, and dramatically suppressed Wnt signaling pathway in vitro and in vivo. Furthermore, we showed that treatment with UNC0638 restores the expression of APC2 expression in these cells through promoter demethylation. Restoring HP1α and silencing APC2 respectively attenuated the inhibitory effects on cell proliferation and Wnt signaling pathway in cancer cells in which G9a was silenced or suppressed.

**Conclusions:**

These findings demonstrate that overexpressed G9a represents a promising therapeutic target, and targeting G9a potentially suppresses growth and Wnt signaling pathway partially through down-regulating HP1α and epigenetically restoring these tumor suppressors such as APC2 that are silenced in NSCLC.

**Electronic supplementary material:**

The online version of this article (10.1186/s12943-018-0896-8) contains supplementary material, which is available to authorized users.

## Background

Lung cancer is the leading cause of cancer-related death worldwide [1]. About 85% of lung cancers are non-small cell lung cancer (NSCLC), while lung adenocarcinoma roughly accounts for about 50% of NSCLC [1]. Although there have been advances in targeted therapies and immunotherapy, the 5-year survival rate for NSCLC patients remains only 15% [2]. Therefore, novel therapeutic approaches are urgently needed for the lethal malignancy.

Recent studies have uncovered an important role for epigenetic changes in tumor progression and treatment resistance [3, 4]. G9a, a histone methyltransferase encoded by the euchromatic histone-lysine N-methyltransferase 2 gene (EHMT2), is a major conserved protein lysine methyltransferase that has a primary role in catalyzing monomethylation and dimethylation of H3K9 (H3K9me1 and H3K9me2), which plays a key role in regulating gene expression and chromosome structure during mammalian development [5, 6]. Studies have revealed that G9a is overexpressed in a number of cancers, including esophageal squamous cell carcinoma, hepatocellular carcinoma, brain cancer, multiple myeloma, and aggressive ovarian carcinoma; and overexpressed G9a is found to be associated with enhanced proliferation and metastasis of various cancer cells [7, 8].

G9a is shown to directly bind DNA methyltransferase 1 (DNMT1), and G9a and DNMT1 colocalize with H3K9me2 on heterochromatic regions of chromosome. The complex of DNMT1 and G9a enhances DNA and histone methylation for stable repression of gene expression [9]. Generally speaking, G9a may participate in carcinogenesis through either suppression of tumor suppressors, such as CDH1/E-Cadherin) [10] and p53 [11], or activation of oncogenic signaling pathways such as hypoxia-response [12] via transcriptional repression of a number of critical tumor suppressors or inhibitors in a histone or non-histone dependent manner in various human cancers [13, 14]. For example, a study showed that G9a is required for TGF-β-induced epithelial-to-mesenchymal transition (EMT) in head and neck squamous cell carcinoma [5]. Another study demonstrated that G9a promoted invasion and metastasis by regulation of the epithelial cellular adhesion molecule (EpCAM) in lung cancer [4]. However, it is still largely unknown about the targeting genes and signaling pathways by which G9a is involved in the disease progression of lung cancer.

Wnt/beta-catenin (β-catenin) signaling pathway promotes cell proliferation, maintains stem cell multipotency, and stimulates EMT in cancer [15, 16]. Inhibition of Wnt signaling pathway has been confirmed to suppress cellular proliferation in NSCLC cell lines, therefore, Wnt signaling pathway is a strong candidate target for therapy [17]. In NSCLC, although β-catenin and adenomatosis polyposis coli (APC) mutations are uncommon, hyperactivation of the Wnt signaling pathway is commonly found. Interestingly, a recent study showed APC2, a homologue of APC, also plays an important role in normal homeostasis and tumorigenesis [18]. Notably, APC2 is frequently inactivated by promoter methylation in lung cancer [19, 20]. Studies have demonstrated that Wnt signaling pathway can be activated by overexpression of Wnt ligands such as Wnt1/2/3 in NSCLC [17]. Epigenetic silencing (eg. promoter hypermethylation) of extracellular Wnt inhibitors, such as secreted Frizzled-related proteins (SFRPs), Wnt inhibitory factor-1 (WIF1), and DICKKOPFs (DKKs), may stabilize β-catenin and lead to accumulation of β-catenin and consequent activation of Wnt signaling pathway [21–23]. Interestingly, a previous study showed that epigenetic silencing of Wnt antagonists SFRP-1 and Axin-2 was associated with H3K9me2 and aberrant Wnt/β-catenin signaling in neuroendocrine tumors of gastrointestinal tract, showing a connection between methylated H3K9 and activation of Wnt signaling pathway [24]. Based on the evidences described above, we hypothesize that targeting G9a may suppress crucial signaling pathways involved in cancer malignancy including Wnt signaling pathway through epigenetically modulate gene expression in NSCLC.

In this study, we first examined aberrant G9a expression and deciphered its transcriptional regulatory network and highlighted its complex role in gene expression, and we found that APC2 was dramatically upregulated upon G9a knockdown, while heterochromatin protein 1 alpha (HP1α), which binds euchromatic loci during the process of gene silencing in cooperation with DNMT1 [25], was significantly downregulated. We further investigated the impact of G9a inhibition on cellular proliferation and Wnt signaling pathway and underlying mechanisms in NSCLC cells. We demonstrated that inhibition of G9a suppressed cellular proliferation and Wnt signaling pathway in NSCLC cells, suggesting overexpressed G9a represents a potential therapeutic target for NSCLC treatment.

## Methods

### Patients selection and clinical data collection

This study was reviewed and approved by the Institutional Review Board (IRB, IRB 12299) of City of Hope National Medical Center. A total of 250 patients with lung adenocarcinoma and squamous carcinoma who underwent surgical resection for curative intent between 2002 and 2014 without preoperative chemotherapy or radiation therapy were included. Tissue microarrays were created using cancer and matched normal tissues.

### Analyzing G9a mRNA expression in NSCLC using publicly available TCGA gene expression data

G9a mRNA expression and survival data for 517/494 lung adenocarcinoma (LUAD)/lung squamous carcinoma (LUSC) and 59/46 normal lung tissues was accessed from the Cancer Genome Atlas (TCGA) public data portal and extracted from expression dataset from Cancer Bioportal (http://www.cbioportal.org/). For data analysis, normalized RNA-Seq data (version 2, level 3) was used as gene expression value and the median was used to classify samples into high and low expression groups. RNA-Seq gene expression data of LUAD and LUSC samples was also downloaded from TCGA data portal to investigate the correlation between the level of G9a mRNA with these selected gene mRNA in the same tissue.

### Cell culture, and proliferation assays

Human lung cancer cell lines: A549 (p53 wild-type, K-Ras mutated), H1299 (p53-null, K-Ras mutated), and H1975 (p53 mutated, K-Ras wild-type, EGFR mutated) cells were purchased from American Type Culture Collection (ATCC). All cells were cultured in DMEM or RPMI medium supplemented with 10% fetal bovine serum (FBS), 100 U/mL penicillin, and 100 mg/mL streptomycin. For proliferation assessment, cells were seeded in 6-well plates in 3 replicates at densities of 2.0 × 10^5^ cells per well, and were monitored at 72 h using the trypan blue exclusion-based viable cell counting method by Vi-CELL® XR Cell Viability Analyzer (Beckman Coulter). UNC0638, a specific inhibitor for G9a [26], was purchased from Sigma-Aldrich (St. Louis, MO). The half maximal inhibitory concentration (IC_50_) of UNC0638 to each cell was also calculated accordingly.

### siRNA transfection, RNA-Seq data analysis, and qRT-PCR

Two independent G9a siRNAs: Cat No.10620318 (5′-GGCAUCUCAGGAGGAUGCCAAUGAA-3′) and Cat No. 10620319 (5′-UUCAUUGGCAUCCUCCUGAGAUGCC-3′) purchased from Thermo Fisher Scientific Corporation (Carlsbad, CA) were applied to silence G9a expression. One APC2 siRNA: Cat No.121574 (5′-CCUACAGGGAAAACUGGAGTT-3′) and HP1α siRNA ID #: s23884 (5′-GCAGAGCAAUGAUAUCGCUtt-3′) were purchased from Thermo Fisher Scientific Corporation to silence APC2 and HP1α expression. A total of 45 pMol siRNA was used for 1 × 10^5^/well cell in a 6-well plate with Lipofectamine RNAiMAX (Invitrogen) to silence G9a expression using the protocol described previously [27]. At 72 h post-transfection, total RNA and protein were extracted for RNA-Seq and Western blot analysis. Transcriptome libraries and RNA sequencing analysis were performed, aligned and normalized according to the Illumina Genome Analyzer II (Illumina, San Diego, CA, USA) manufacturer’s instruction with minor modifications as we described previously [28]. Both gene set enrichment analysis (GSEA) and database for annotation, visualization and integrated discovery (DAVID) were used to analyzed the Gene Ontology and canonical pathways significantly modulated by the knockdown of G9a [29, 30]. Quantitative reverse transcription polymerase chain reaction (qRT-PCR) was carried out in the ABI Prism 7900HT Sequence Detection System (Applied Biosystems) using cDNA synthesized from 1.0 μg of total RNA by the Super-script III first-strand cDNA synthesis Kit (Invitrogen) and SYBR Green PCR Master Mix to quantitatively measure mRNA expression of selected genes with gene specific primers, sequences are available upon request. Data were presented as the relative quantity of targets, normalized with respect to internal control, or relative to a calibrator sample.

### TOP luciferase reporter assay and expression of HP1α

NSCLC cells were first transfected with either control or G9a specific siRNA at a concentration of 5.0 × 10^4^ cells per well on 24-well plates. At 24 h post-transfection, cells were further transfected with 125 ng of T-cell factor (TCF)/LEF-1 reporter plasmid plus 5 ng *Renilla* luciferase reporters using Lipofectamine™-2000. Forty hours after reporter plasmid transfection, cells were treated with or without 100 ng/ml Wnt3a for another 8 to 12 h, firefly and *Renilla* luciferase activities were determined and calculated as described previously [28]. All experiments were done in triplicate. The pcDNA HA-tagged HP1α was a gift from Naoko Tanese (Addgene plasmid # 24078) [31], and it was transfected into cells using Lipofectamine™-2000 to rescue HP1α expression.

### Western blot, immunohistochemistry, and immunofluorescence

The antibodies against Actin, APC2, DKK1, EpCAM, G9a, H3K9-Me2, HP1α, and WIF1, p53, c-Myc were purchased from Cell Signaling Technology, Abcam, Santa Cruz Biotechnology or GeneTex respectively. Immunohistochemistry **(**IHC) was performed using anti-G9a antibody from GeneTex as described previously [27]. Expression levels of G9a in all clinical samples were scored based on the percentage of positively stained cells as described previously [27]. G9a IHC staining was graded as negative (0), if &lt; 1% cells displayed positive nuclear staining. Those cancer tissues with 1–4%, 5–25%, or &gt; 25% of cancer cells positive staining for G9a protein were graded as 1+, 2+, or 3+ respectively [27]. After cells on gelatin-coated glass coverslips were first transfected with either control or G9a siRNA and stimulated with or without as described above, subconfluent cells were fixed, permeabilized, blocked and incubated with anti-G9a antibody (Abcam, 1:500 dilution), and then imaged as described previously [28].

### Treatment of xenograft with G9a inhibitor UNC0638

All animal protocols were performed in the animal facility at City of Hope National Medical Center accordance with federal, local, and institutional guidelines. NOD/SCID/IL2Rgamma null mice (NSG) mice (Jackson Labs, Bar Harbor, ME; 24–27 g, 6–8 weeks of age) were used for xenograft experiment. A suspension of 5 × 10^6^ tumor cells (H1299) in 0.1 ml RPMI 1640 was mixed with 0.1 ml BD Matrigel™ (BD Science) and injected into the subcutaneous dorsa of mice at the proximal midline. When the tumor volume was 90–110 mm^3^, mice were randomized. Mice treatment with UNC0638 was performed by continuous administration of 100 μl of 5 and 10 mg/ml of UNC0638 intraperitoneal (i.p.) injection via mini-osmotic pump (ALZA, Palo Alto, CA) as described previously [32]. These pumps (internal volume, 100 μl) continuously deliver test agents at a rate of 0.25 μl/h for 14 days. The control group received comparable i.p. implanted, vehicle-loaded pumps. The pump was implanted i.p. under sterile conditions after a small midline incision. The mice were weighed and tumors were measured and weighed using standard protocols [32].

### DNA methylation analysis

Genomic DNA was extracted using QIAamp DNA Mini Kit (Qiagen). A total of 1.5 μg of genomic DNA were modified using sodium bisulfite to deaminate selectively unmethylated cytosine residues to uracil, while 5-methyl cytosine residues were not modified. The bisulfite modification was performed using the EZ DNA Methylation Kit™ (Zymo Research, Orange, CA, USA), and 40 ng of modified DNA was used per PCR amplification. A forward (5′-GGGTYGTTATTGGTTGTTGTTATGG-3′) and a reverse (5′-AAACRCCTAAATCTAAAACCTCCTC-3′) primers specific for the bisulfite-converted DNA were used to amplify a highly methylated CpG island (from − 2840 to − 2560, encompassing ~ 35 CpGs) in the APC2 gene promoter region [19]. And the amplified PCR product was sequenced using sequence primer (5′- ATTGGTTGTTGTTATGGTATTAGTT-3′). Based on the percentage of methylated, a CpG dimer was assessed as methylated, if the percentage of methylated CpG was &gt; 60%; a CpG was assessed as unmethylated, if the percentage of methylated CpG was &lt; 60%.

### Statistical analysis

All experiments were performed in duplicates or triplicates and repeated at least two times in each experiment. Two group comparisons were analyzed for variation and significance using a Student’s *t-*test or Pearson χ^2^ test. All data shown are mean ± s.d. Statistical significance was set at *P &lt;* 0.05. Pearson’s correlation coefficient was used to measure correlation of gene coexpression.

## Results

### Upregulation of G9a mRNA and protein in NSCLC tissues

**Fig. 1:**
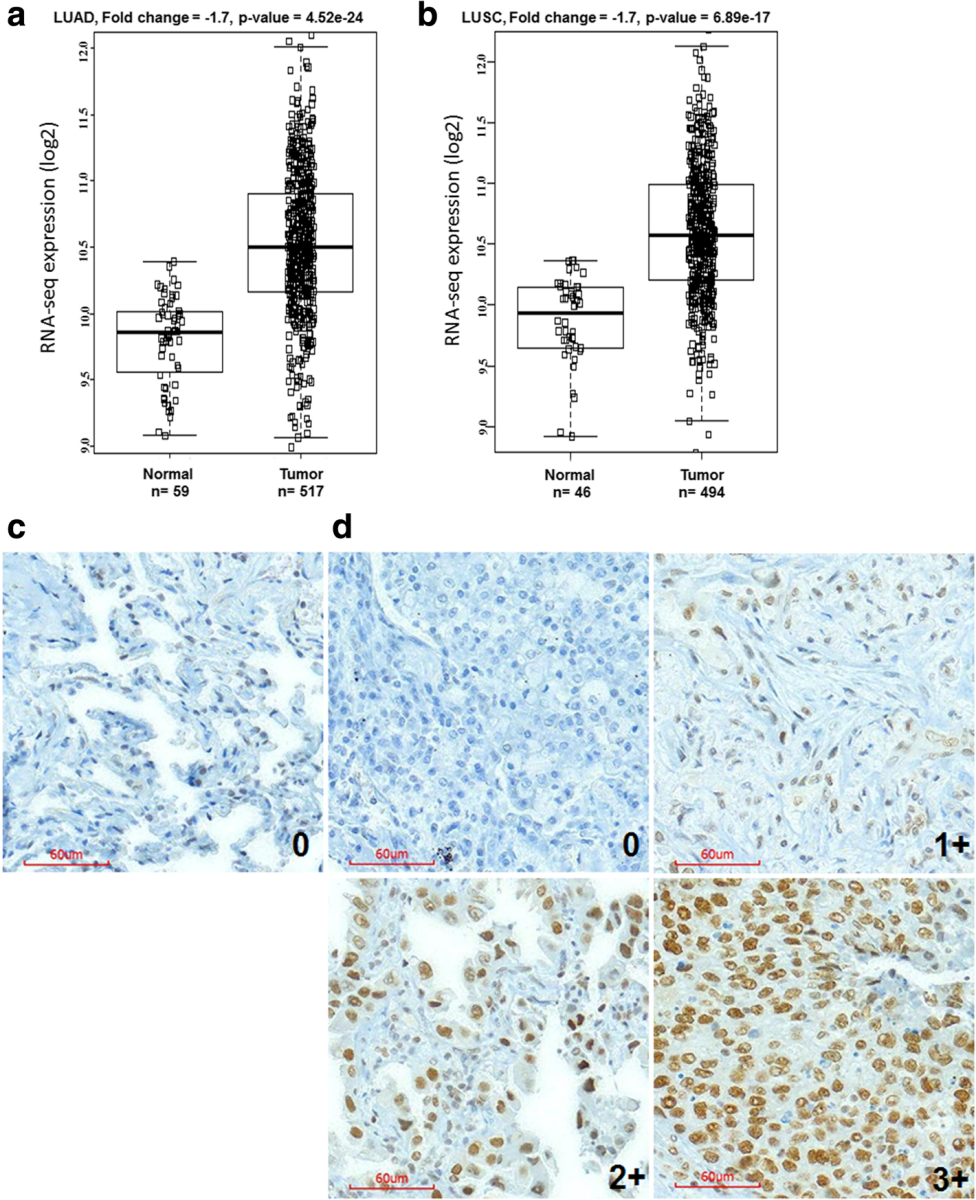
Overexpression of G9a in NSCLC. Box-and-Whisker plot of G9a mRNA expression in **a** lung adenocarcinoma (LUAD), **b** lung squamous carcinoma (LUSC). Box represents first and third quartiles, thick band is median value, and bars extend to ± the interquartile range divided by the square root of the number of samples) were applied to describe G9a gene expression values. Compared to normal lung tissues (Gr1, *n* = 59/46), G9a mRNA was significantly increased in LUAD/LUSC (Gr2, *n* = 517/494) (fold change/FC = 1.7, and *P =* 4.52E-24/6.89E-17). **c** Photomicrographs of G9a in normal lung tissue. **d** Photomicrographs of G9a in four representative lung adenocarcinoma sections scored as 0 to 3+ (magnification, × 200; red bar, 60 μm)

In the present study, we first mined lung cancer gene expression data from TCGA to examine the level of G9a mRNA in NSCLC tissues. As shown in Fig. 1, Box-and-Whisker plot shows that G9a mRNA level was moderately variable in NSCLC tissues, and it was substantially higher in at least half of both LUAD N( *N* = 517; Fig. 1a) and LUSC N( N = 494; Fig. 1b) samples in comparison to normal lung tissues (*N* = 105). Importantly, statistical analysis revealed that in comparison to corresponding normal lung tissues, G9a mRNA was significantly higher in NSCLC samples (ratio to normal = 1.7, *P* &lt; 0.01).

To further investigate the G9a protein in NSCLC, we examined G9a protein in about 213 cancer tissue samples by IHC analysis. The results show that G9a protein in normal lung tissues was generally absent or much lower compared to NSCLC tissues. Representative G9a immunostaining images of normal lung tissue (Fig. 1c) and NSCLC tissues (Fig. 1d) reveal a dominantly nuclear staining pattern in both normal and tumor tissues. IHC analysis showed that G9a was absent (scored as 0) in 38.0% (81/213), while G9a levels in 18.8% (40/213), 28.2% (60/213), and 15.0% (32/213) of NSCLC cases were scored as 1 +, 2 +, 3 respectively.

### Changes in gene expression profile in lung cancer cells upon knockdown of G9a

**Fig. 2:**
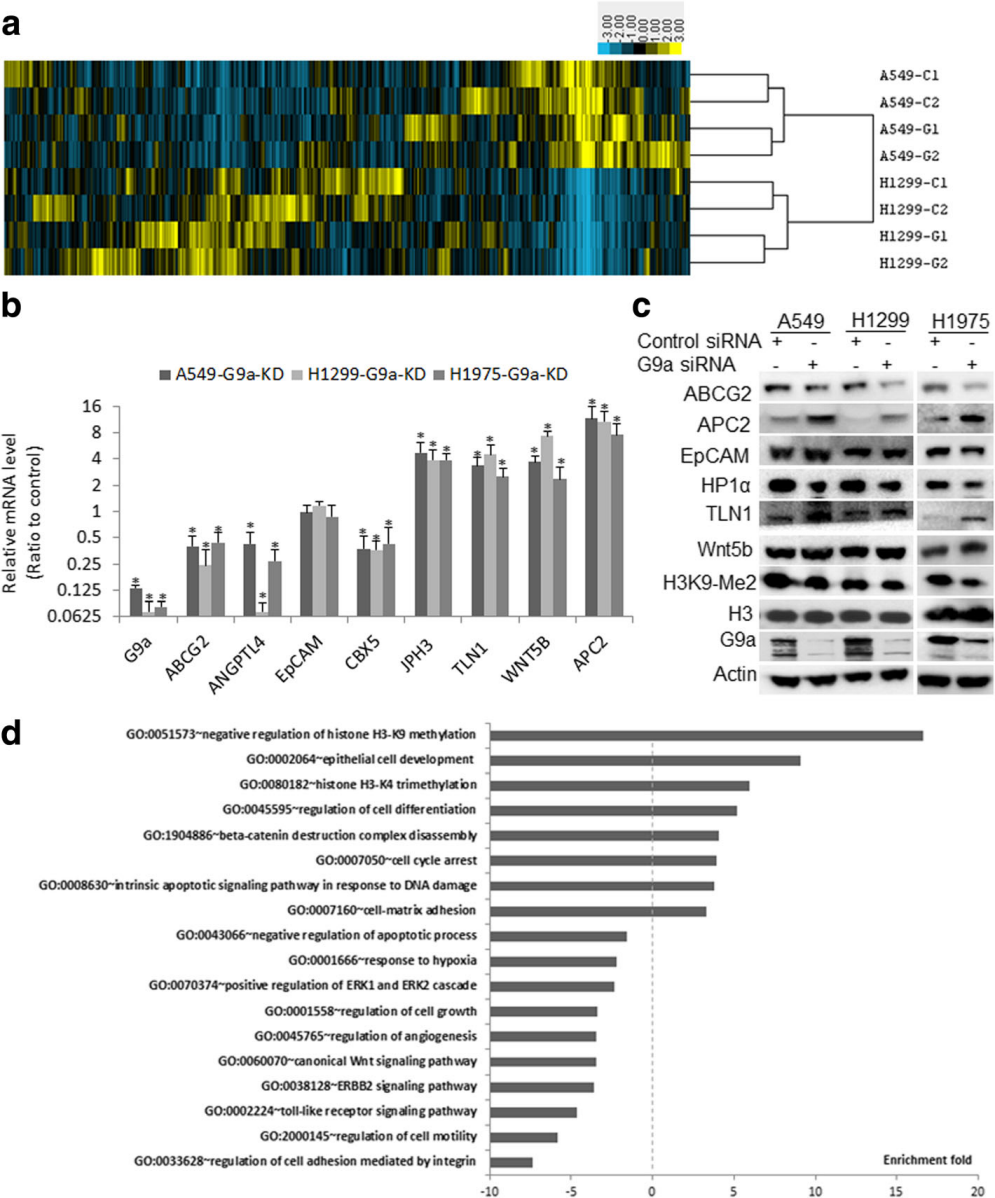
Changes of gene expression profile and gene set in NSCLC cells upon knockdown of G9a. **a** Unsupervised hierarchical clustering analysis of global expression profiles in A549 and H1299 cells upon G9a knockdown. The two top rows represent two independent siRNA repeats, C1/2 is for control siRNA and G1/2 is for specific siRNA1/2 for G9a. **b** qRT–PCR, **c** Western blot analysis for selected genes in A549, H1299, and H1975 cells. The level of each gene in cancer cell transfected with G9a siRNA is the average ratio of triplicates of two repeated experiments, and is presented as the ratio to control sample transfected with scramble siRNA (* *P* &lt; 0.05, compared to control cells transfected with scramble siRNA). **d** Selected gene sets that were enriched upon G9a knockdown in these lung cancer cells

The effect of G9a on global gene transcription is not known in lung cancer. RNA sequencing (RNA-Seq) analysis was utilized to characterize global gene expression change upon knockdown of G9a in A549 and H1299 NSCLC cells. Unsupervised hierarchical clustering analysis of RNA-Seq data depicts the changes in global gene expression profile in these lung cancer cells upon knockdown of G9a (Fig. 2a). G9a knockdown significantly modulated about 8–12% of transcribed genes (1100–1300 genes) in each of these cells; with near 60% of them being down-regulated (Fig. 2a, yellow to blue color) and 40% being up-regulated (Fig. 2a, blue to yellow color), suggesting that H2K9 methylation can both promote and suppress gene transcription. The original RNA-Seq data is saved on NCBI GEO website with accession number GSE113493. Additional file 1: Table S1 lists the differentially expressed genes in two lung cancer cells. Remarkably, knockdown of G9a had pronounced effects on the expression of important cancer-associated proteins including HP1α encoded by Chromobox 5 (CBX5), ATP-binding cassette sub-family G member 2 (ABCG2), angiopoietin-like 4 (ANGPTL4), APC2, urokinase plasminogen activator (u-PA), Junctophilin-3 (JPH3), Talin 1 (TLN1), and telomerase reverse transcriptase (TERT) etc. that are involved in cell proliferation, invasion, and metastasis, drug resistance. Differential gene expression of these selected genes upon knockdown of G9a in A549, H1299, and H1975 were validated by qRT–PCR (Fig. 2b) and Western Blot (Fig. 2c). Using GSEA, we identified multiple gene sets from the Kyoto Encyclopedia of Genes and Genomes (KEGG) that were significantly enriched in lung cancer cells upon G9a knockdown (Fig. 2d) (*P* &lt; 0.05). The majority of gene sets were down-regulated, and important sets in two lung cancer cells were these involved in cell proliferation, migration, and regulation of apoptosis, angiogenesis, response to hypoxia, canonical Wnt, and toll-like receptor signaling; while adhesion to matrix, response to DNA damage, regulation of epithelial cell differentiation, and histone H3-K9 methylation, were significantly enriched in both A549 and H1299 cells (Fig. 2d), indicating that these pathways are potentially involved in G9a-mediated disease progression lung cancer.

### Targeting G9a inhibits proliferation, induces cell cycle arrest in NSCLC cells

**Fig. 3:**
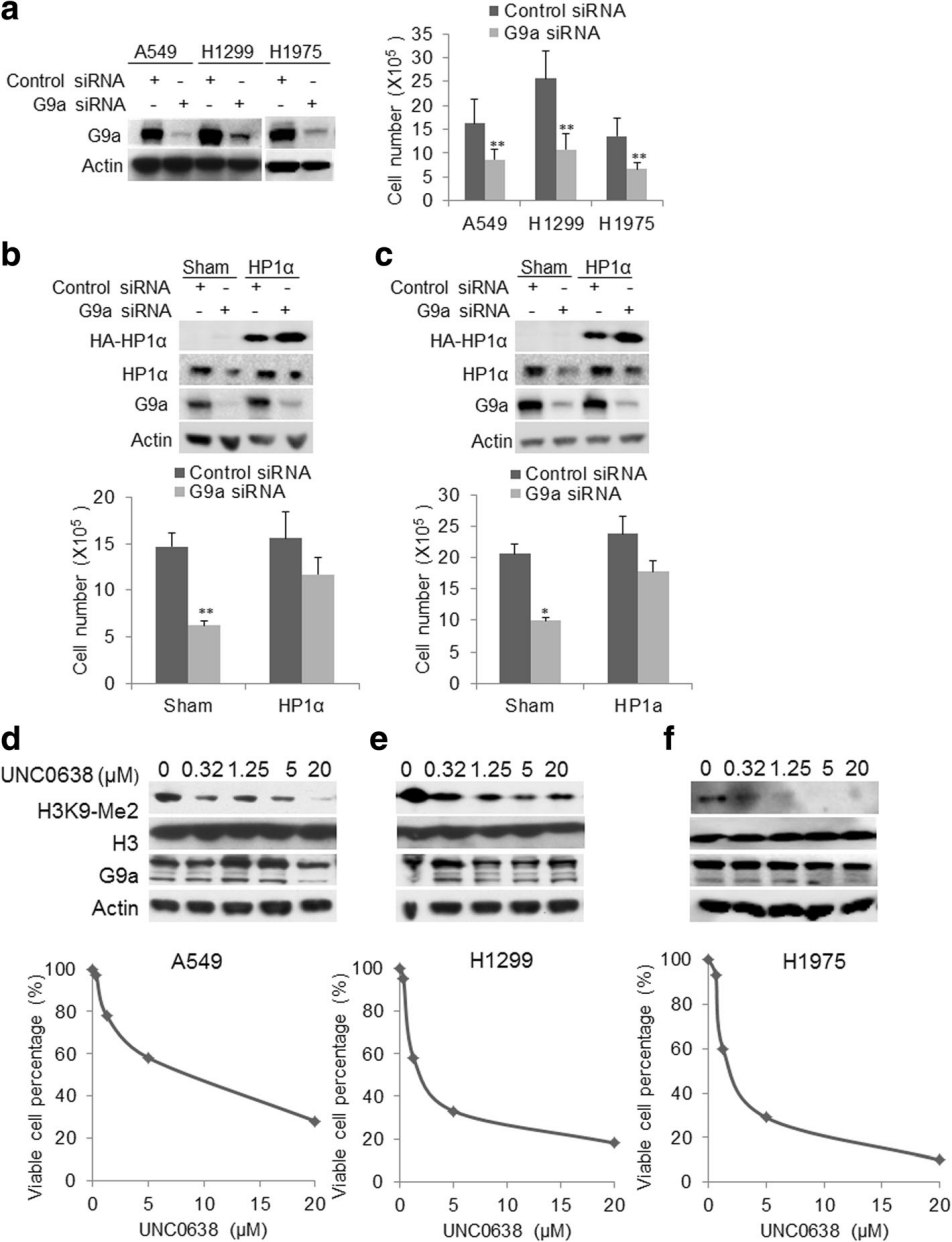
Impact of G9a suppression on in vitro proliferation of NSCLC cells. **a** Western blot analysis (left panel) of cell lysates of A549, H1299, and H1975 cells transfected with either G9a siRNA or control siRNA and harvested at 72 h post transfection. Decrease in cell proliferation (right panel) upon knockdown of G9a (at 72 h post-transfection, *P* &lt; 0.05 compared to control siRNA transfected). Restoration of HP1α expression in **b** A549 (upper panel), **c** H1299 (upper panel) partially abolished inhibitory effects of G9a knockdown on cell proliferation (down panel, *P* &lt; 0.05 compared to sham-transfected cells). Effect of G9a inhibitor UNC0638 on H3K9Me2 (Upper panel for Western blot analysis, H3 for total H3 protein) the proliferation curve (lower panel) of **d** A549, **e** H1299, and **f** H1975 treated with defined UNC0638 for 72 h. *P &lt; 0.05, ** *P* &lt; 0.01 compared to control cells

We used siRNA to silence G9a to test its impact on in vitro cellular proliferation in three NSCLC cell lines. As shown in Fig. 3a left panel, G9a siRNA significantly decreased the levels of G9a and H3K9-Me2 protien, knockdown of G9a resulted in a significant decrease of in vitro cellular proliferation in three NSCLC cells over 72 h post-transfection of G9a siRNA in comparison with control cells transfected with scramble siRNA (Fig. 3a right panel, *P &lt; 0.05*). In addition, we found that knockdown of G9a induced cell cycle arrest of A549 in the G1/S phase (Additional file 2: Figure S1a) or H1299 cells in G2/M phase (Additional file 2: Figure S1b). A previous study showed that G9a negatively regulates p53 activity in cancer cells. Herein, we found that knockdown of G9a slightly upregulated wild-type p53 protein in A549 cells, but significantly inhibited the proliferation of all three NSCLC cells regardless of their p53 status (Additional file 2: Figure S2), indicating this regulation may be not critical to proliferation of NSCLC.

Interestingly, RNA-Seq analysis showed that knockdown of G9a downregulated HP1α in these NSCLC cells. Study demonstrated that overexpression of HP1α correlated with enhanced cell proliferation and poor clinical outcomes of breast cancer [33]. Therefore, we tested if downregulation of HP1α contributed to the inhibition of cell proliferation seen when G9a was silenced, and found that knockdown of HP1α by siRNA significantly suppressed in vitro proliferation of A549, H1299 and H1975 cells (Additional file 2: Figure S3). Notably, we also observed that overexpression HP1α itself promoted cell proliferation and restoration of HP1α expression in A549 (Fig. 3b, upper panel) and H1299 (Fig. 3c, upper panel) partially abolished inhibitory effects of G9a knockdown on cell proliferation (Fig. 3b-c, down panel, *P* &lt; 0.05 compared to sham-transfected cells). To further test if a small molecular inhibitor of G9a could suppress NSCLC proliferation, we treated three lung cancer cells with a selective G9a inhibitor UNC0638 [26]. UNC0638 treatment dramatically decreased H3K9Me2 protein level (upper panel) and cellular proliferation (lower panel) of A549 (Fig. 3d), H1299 (Fig. 3e) and H1975 (Fig. 3f) in a dose-dependent manner. The IC_50_ of UNC0638 was about 5.0, 2.5, and 3.5 μM for A549, H1299, and H1975 respectively. Overall, these data indicated that targeting G9a by siRNA or small molecule inhibitor significantly suppressed in vitro proliferation of NSCLC cell lines.

### Inhibition of G9a suppresses Wnt/β-catenin pathway in NSCLC

**Fig. 4:**
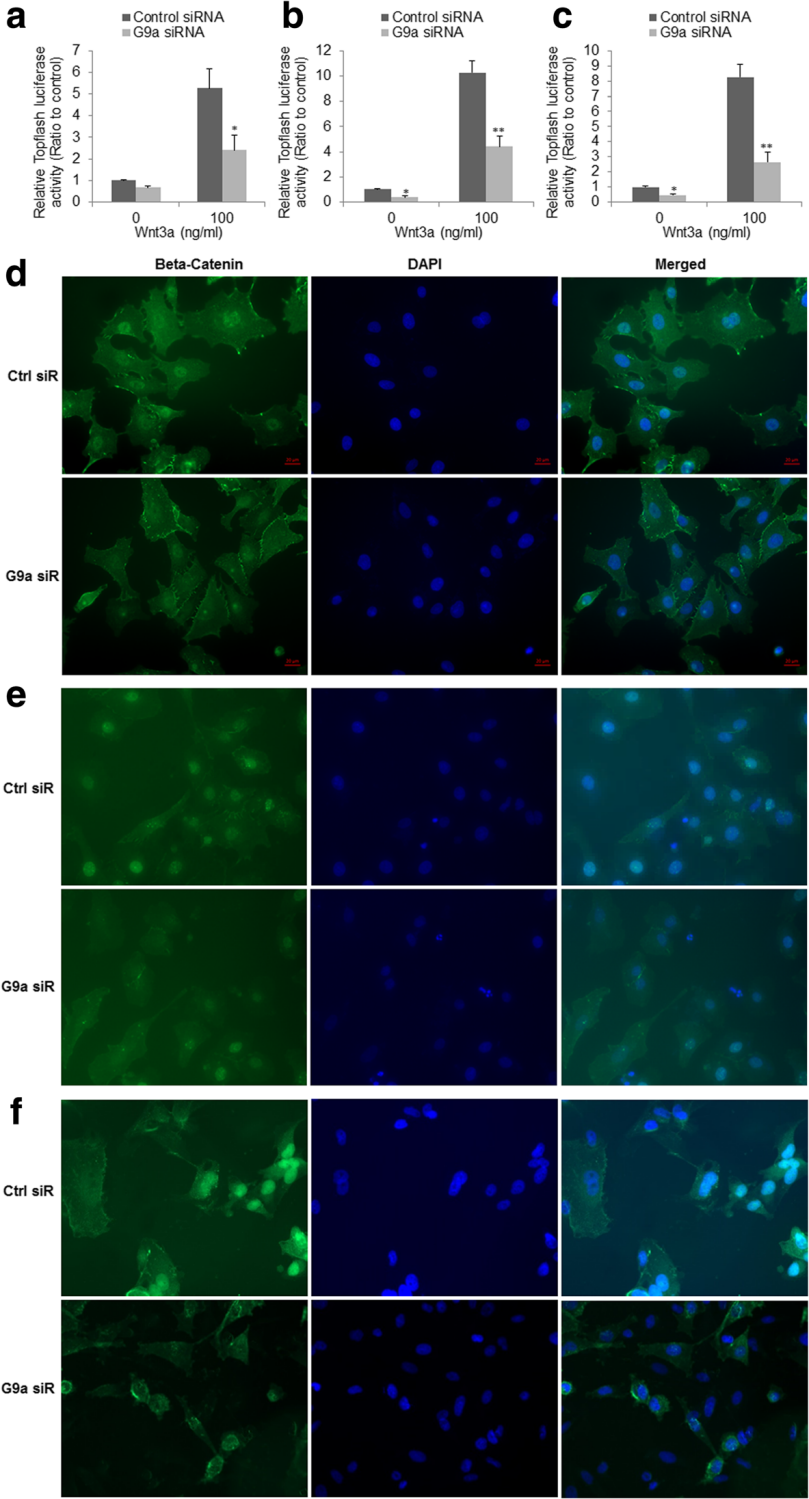
Inhibition of Wnt signaling pathway upon knockdown of G9a in NSCLC cells. Quantitation of TOPFlash luciferase reporter activity in **a** A549, **b** H1299, and **c** H1975 transfected with control or G9a siRNA as indicated (**P &lt; 0.01, compared to control siRNA). Double-label fluorescent immunohistochemistry of cells as indicated at 72 h post-transfection with G9a or control siRNA in **d** A549, **e** H1299, and **f** H1975 cells. Blue is for DAPI nuclear stain, and green is for β–catenin stain

By using RNA-Seq and GSEA analysis, we found that knockdown of G9a enriched β–catenin destruction complex disassembly and down-regulated Wnt signaling pathway, indicating targeting G9a may suppress Wnt signaling pathway. We further investigated the effect of G9a knockdown on the Wnt signaling pathway in NSCLC cells. As β-catenin is an obligatory cofactor for T cell factor (TCF), increase in TCF transcriptional activity indicates upregulation of β-catenin. We first measured TOPFlash-Luciferase (TCF Reporter Plasmid, Millipore) reporter activity [27], and examined the level of activated Wnt signaling pathway using nuclear accumulation of β-catenin upon G9a knockdown in all three cells. TOPFlash-Luc reporter assays demonstrated that knockdown of G9a significantly suppressed both basic and the induced TCF transcriptional activity by Wnt3a, a ligand/activator of Wnt signaling pathway in A549 (Fig. 4a, *P* &lt; 0.05), H1299 cells (Fig. 4b, *P* &lt; 0.05) and H1975 cells (Fig. 4c, *P* &lt; 0.05). In agreement with the TOPFlash-Luc assay, double-label fluorescent immunohistochemical analysis showed that accumulation of nuclear β-catenin was relatively lower in cells without Wnt3a stimulation (data not shown), however, the accumulation was dramatically elevated in cells upon Wnt3a stimulation (Fig. 4d-f). Knockdown of G9a decreased the accumulation of nuclear β-catenin especially in these cells stimulated by Wnt3a in A549 (Fig. 4d), H1299 (Fig. 4e), and H1975 (Fig. 4f) cells. Interestingly, slight decrease of β-catenin was also observed in these three cells transected with G9a siRNA. Taken together, these results suggest that knockdown of G9a can suppress Wnt signaling pathway.

**Fig. 5:**
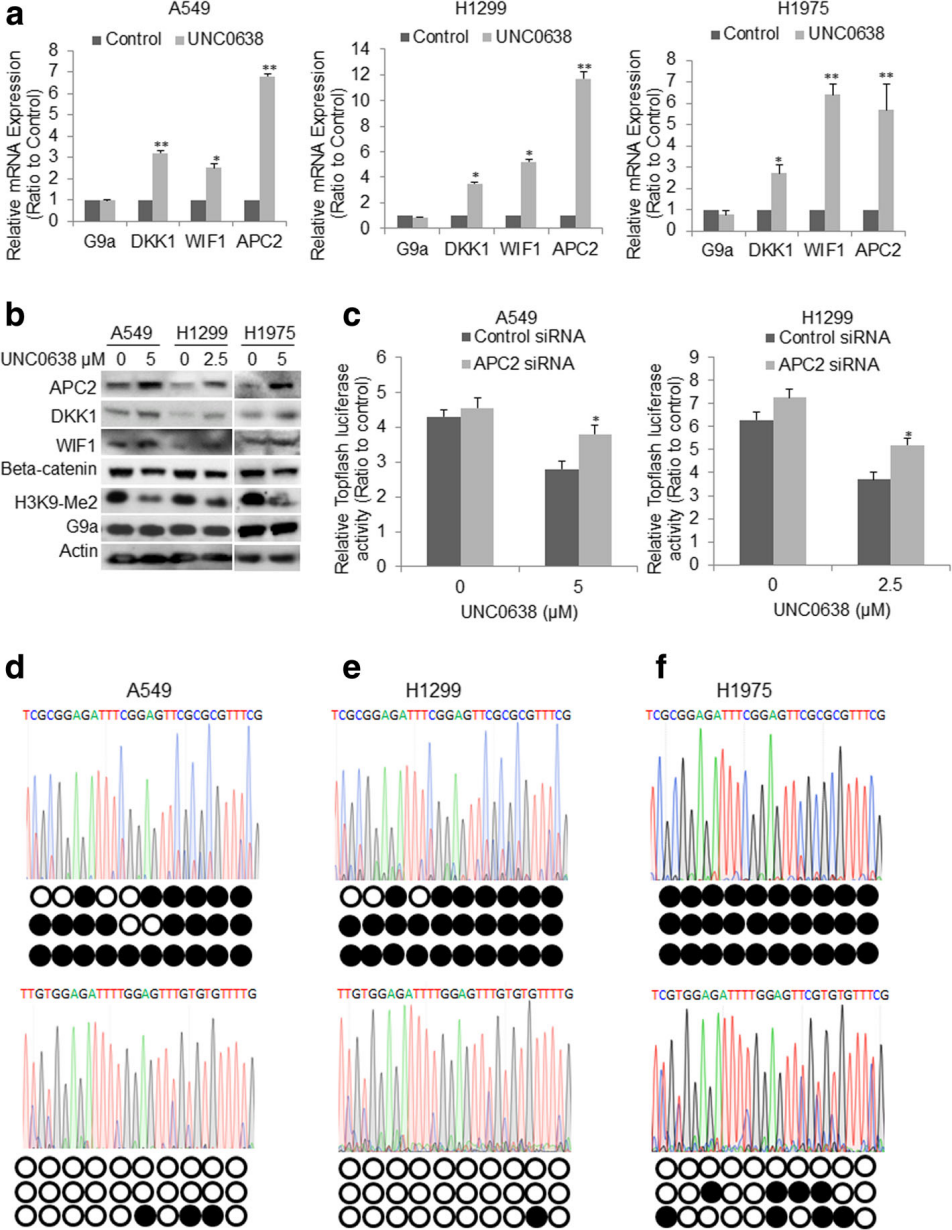
Impact of UNC0638 treatment on Wnt activation and downstream molecules. **a** qRT–PCR for selected genes in A549 (left panel), H1299 (middle panel) and H1975 (right panel). Data is presented as the ratio to control sample (*P &lt; 0.05, **P &lt; 0.01, compared to control siRNA). **b** Western blot analysis of G9a, H3K9-Me2, APC2, DKK1, WIF1, and beta Catenin in the three cells treated with UNC0638 for 72 h. **c** TOPFlash-Luc reporter assays showed that knockdown of APC2 partially attenuated the inhibitory effect of UNC0638 on Wnt3a-induced reporter activity in both A549 cells (left panel) and H1299 cells (right panel). Bisulfite-PCR sequencing chromatogram for the representative CpG dimers and the methylation status (the black dots represent for methylated, the white for unmethylated CpG dimers) of the 30 CpG dimers in the APC2 promoter region (− 2840~ − 2560) of **d** A549, **e** H1299, and **f** H1975 cells. The upper panel of **d**-**f** is for cancer cells treated with vehicle, the lower panel is for cancer cells treated with UNC0638

### Suppression of Wnt activation through de-repressing APC2 via promoter demethylation by UNC0638 treatment in NSCLC cells

By RNA-Seq, we found that APC2, which forms a destructive complex capable of binding β-catenin to function as an important inhibitor of Wnt signaling pathway [34], was dramatically upregulated upon G9a knockdown, suggesting that targeting G9a would restore these genes silenced by epigenetic machinery. To test this hypothesis, we suppressed G9a in A549, H1299, and H1975 cells by UNC0638 treatment, and quantitative RT-PCR (Fig. 5a**)** analysis showed that APC2, DKK1, and WIF1 gene were significantly upregulated. Western blot analysis confirmed that UNC0638 treatment dramatically reduced the level of H3K9Me2, increased the levels of APC2, DKK1, and WIF1 proteins, and resulted in decreased β-catenin in these cancer cells (Fig. 5b). Considering the important roles of these inhibitors in Wnt signaling pathway, we hypothesized that restoring these Wnt inhibitors suppress Wnt signaling pathway by targeting G9a. To prove this, we silenced APC2 to examine the inhibitory effect of UNC0638 on activation of Wnt signaling pathway stimulated by Wnt3a in A549 and H1299 cells. TOPFlash-Luc reporter assays showed that UNC0638 treatment significantly suppressed the luciferase activity in both A549 (Fig. 5c**,** left panel) and H1299 cells (Fig. 5c, right panel), and knockdown of APC2 by siRNA transfection partially attenuated the inhibitory effect of UNC0638 on Wnt3a-stimulated reporter activity in these two cells, indicating upregulation of APC2 contributed to Wnt inhibition upon suppression of G9a. In addition, to further explore the correlation between G9a mRNA with APC2, DKK1, and WIF1 mRNA expression in NSCLC tissues, we downloaded global gene expression RNA-Seq data of NSCLC tissues from TCGA gene expression data portal. The correlation analysis showed that G9a mRNA level was inversely correlated with DKK1 (Additional file 2: Figure S4a, *P* = 0.00828, *R* = − 0.38) and WIF1 (Additional file 2: Figure S4b, *P* = 0.0403, *R* = − 0.3) in 46 of lung cancer tissues, and a slight reverse but insignificant correlation between APC2 and G9a was observed in lung cancer tissues (data not shown).

Using RNA-Seq analysis, we found that besides APC2 other genes including ICAM5, JPH3, MGMT, MMP2, and RASSF1 whose expression are epigenetically silenced, were also upregulated with G9a inhibition (Additional file 2: Figure S5) [19, 35]. To explore how G9a inhibition upregulated APC2 gene expression, we examined the promoter methylation changes of APC2 in A549, H1299, and H1975 cancer cells treated with UNC0638. Bisulfite-PCR sequencing analysis revealed that 24 out of 30 CpG dimers in the APC2 promoter region (− 2840~ − 2560) of A549 cells (Fig. 5d), 27 out of 30 CpG dimers in that region of H1299 cells (Fig. 5e), and all of 30 CpG dimers of H1975 cells (Fig. 5f) were methylated (the upper sequencing chromatogram for methylation status of selected CpGs, black dot for methylated and white for unmethylated CpG). Notably, UNC0638 treatment significantly decreased methylated CpGs in the APC2 promoter region of A549 cells (Fig. 5d, the lower panel), H1299 cells (Fig. 5e, the lower panel), and H1975 (Fig. 5f, the lower panel). These data suggested that UNC0638 may restore these silenced genes’ expression through promoter demethylation in non-small cell lung cancer cells. In addition, a recent study demonstrated that targeting G9a inhibited breast cancer growth and markedly downregulated c-Myc, a key oncogenic component of the Wnt signaling pathway [36]; we also examined the effect of knockdown of G9a on c-Myc protein in these NSCLC cells with moderate to high level of c-Myc protein, and found that knockdown of G9a did not substantially downregulate c-Myc expression in NSCLC cells (Additional file 2: Figure S3), suggesting c-Myc may be not essential to G9a-mediated Wnt activation in NSCLC. Taken together, the above data indicate that G9a may contribute to lung cancer cellular proliferation partially through transcriptional suppression of Wnt inhibitors expression.

### UNC0638 suppressed xenograft tumor growth and Wnt signaling pathway in NSG mouse

**Fig. 6:**
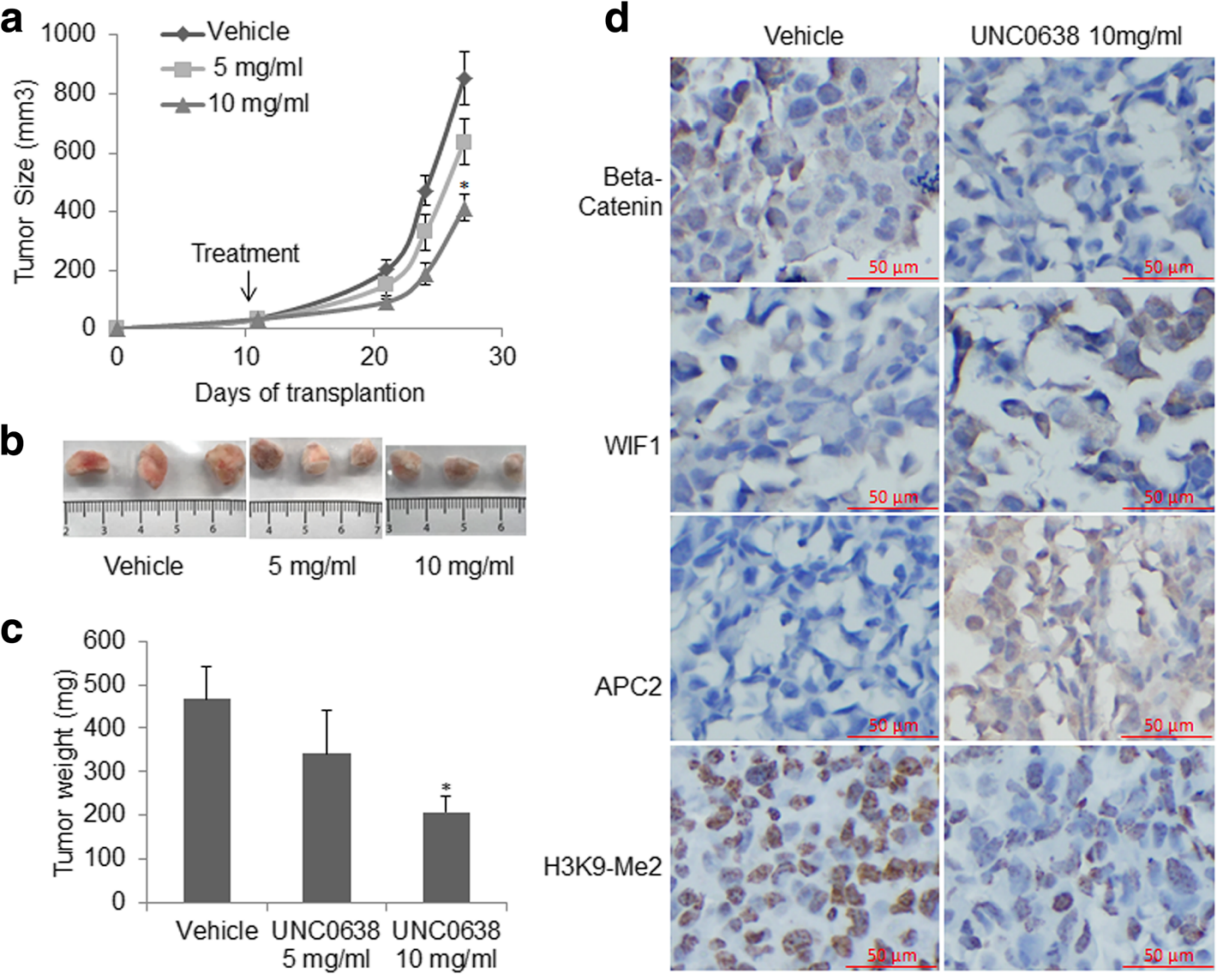
Treatment of H1299 xenografts with UNC0638 strongly suppressed tumor growth and Wnt signaling pathway. **a** Tumor growth curves show UNC0638 treatment significantly inhibited H1299 xenografts growth. Xenograft growth was measured in vivo during 28 days, and treatment started at 10 days post-transplantation. **b** Resected tumors (three replicates) from different treatment regimens were illustrated for growth response to UNC0638 treatment (upper for vehicle, lower left and right for 5 and 10 mg/ml UNC0638). **c** Tumor weight in the mice treated with control (vehicle) and UNC0638 (*P &lt; 0.05, compared to controls). **d** IHC stains for H3K9-Me2, APC2, and WIF1 in these xenograft tissues. IHC analysis shows that treatment with UNC0638 strongly increased APC2 and WIF1 proteins, concurrently decreased H3K9-me2 and β–catenin (magnification, × 200; red bar, 50 μm)

We tested the in vivo anti-tumor effect of the G9a inhibitor UNC0638 in mouse xenograft models. H1299 cancer cells were used to establish subcutaneous xenograft tumors in NSG mice. Compared to the solvent control, treatment with UNC0638 via mini-osmotic pump at the dose of 10 mg/kg significantly suppressed xenograft tumor growth (*P* = 0.05; Fig. 6a**)**. The average tumor size in the mice treated with UNC0638 on day 28 after the implantation was significantly lower than the tumor weight in the mice treated with PBS (Fig. 6b-c). These results revealed that UNC0638 significantly inhibited tumor growth in a NSG xenograft model. The xenograft tissues were fixed and analyzed by IHC to investigate the effect of UNC0638 treatment on Wnt signaling in vivo. As shown in Fig. 6d, compared to the solvent control, treatment with UNC0638 strongly decreased the level of H3K9-Me2 protein. Consistent with in vitro data, UNC0638 treatment dramatically restored APC2 and moderately increased WIF1 protein in the xenograft tissues. Concurrently, β–catenin was decreased in the xenograft tissues treated with UNC0638, indicating a suppression of Wnt signaling pathway activation.

## Discussion

Recently, studies have revealed that G9a plays an important role in regulating gene expression and chromosome structure, and elevated G9a has been identified in many types of human cancers, and are associated with enhanced proliferation, invasion, metastasis, advanced stage, and poor clinical outcome of cancers [5–8, 13, 14, 37–39]. G9a, forming a heterodimeric complex with G9a-like protein (GLP), is functionally responsible for methylating H3K9, and then methylated H3K9 serves as a binding platform for HP1, and HP1 directly interacts with and activates DNMT1 activity to permanently silence gene expression [9, 38, 40]. Notably, apart from histones, G9a has also been found to interact with other proteins. G9a can suppress p53 [11], enhance the transcriptional activity of HIF-1α [12]. The tumor suppressor p53 plays an important role in cell cycle control. We herein found that G9a inhibition significantly suppressed the proliferation of NSCLC cells regardless of p53 status, indicating this regulation may be not critical to NSCLC proliferation. A study showed that G9a also inhibits E-cadherin and is required for TGF-β-induced EMT in head and neck squamous cell carcinoma tumorsphere formation [41]. Therefore, G9a may contribute to malignancy through various molecules and mechanisms in cancers.

Methylation of H3K9 is normally associated with gene silencing [42]. A previous study showed that G9a promoted lung cancer invasion and metastasis by silencing the cell adhesion molecule EpCAM, and knockdown of G9a in fast-growing cell lines led to a less aggressive phenotype [38]. We only observed a minimal upregulation of EpCAM in two of three NSCLC cells upon G9a knockdown, indicating other molecular mechanisms and substrates through which G9a may promote carcinogenesis in NSCLC. In fact, more G9a-regulated genes including CDH1, DSC3, DUSP5, and SPRY4 have been recently identified [10, 43]. The majority of these genes function as tumor suppressors in different cancers. We found genes including HP1α, multidrug resistance gene ABCG2 [44], ANGPTL4, APC2, u-PA, JPH3, TLN1, and TERT, and signaling pathways involved in cellular differentiation, growth, adhesion, angiogenesis, hypoxia, apoptotic, canonical Wnt, and toll-like receptor signaling pathways were differentially expressed or significantly altered in lung cancer cells upon G9a knockdown. A study showed that G9a knockdown in breast cancer changed a cohort of genes involved in EMT, a phenotypic conversion linked with metastasis [43]. In their study, they found epithelial markers such as claudin and E-cadherin were upregulated after G9a depletion, whereas mesenchymal markers, including N-cadherin and vimentin, were downregulated. Consistent with previous studies, we also showed that knockdown of G9a significantly suppressed cell proliferation in human NSCLC cell lines [26, 45–47]. Inconsistent with their findings [43], we did not observe a pronounced change in expression of these EMT markers. These findings suggest that G9a may differentially regulate gene expression in different cell contexts.

HP1 binds specific euchromatic loci in mammals during the process of gene silencing in conjunction with methylated H3K9 by G9a, however, mechanism of action of HP1 at euchromatic loci is not well understood [25]. A previous study showed that the levels of all isoforms of HP1 were reduced after HDAC inhibition [48], indicating complex interactions between these chromatin modulators. Interestingly, we herein found that both mRNA and protein level of HP1α were down-regulated upon G9a knockdown in these two cells, and we further found that G9a inhibitor UNC0638 significantly decreased HP1α that was accompanied by decreased H3K9-Me2 (Additional file 2: Figure S6), indicating HP1α may be also epigenetically regulated by H3K9Me2 and G9a, although the underlying mechanisms remain to be identified. A previous study demonstrated that overexpressed HP1 showed a significant correlation with disease progression and metastasis in breast cancer, and HP1α levels were associated with increased cell proliferation [33]. We also observed that restoration of HP1α expression partially abolished inhibitory effect of G9a knockdown on cell proliferation, suggesting part of the effect of G9a on cancer cell proliferation was mediated by HP1α expression.

We found that targeting G9a suppressed Wnt signaling pathway in NSCLC cell lines. The Wnt signaling pathway is a strong candidate target for therapy as it is aberrantly expressed in most lung cancers and impacts NSCLC tumorigenesis, prognosis, and resistance to therapy [17]. Studies have shown that Wnt signaling pathway can be enhanced by repression of expression of these Wnt inhibitors such as WIF1, SFRP, DDK1 through both genetic and epigenetic alterations in human cancers [24, 42]. Notably, expression of WIF1 is suppressed by promoter hypermethylation of WIF1 in NSCLC cells and tissues [21], and restoring WIF1 expression inhibits lung cancer cell growth [15]. We found that APC2, which forms a destruction complex capable of binding β-catenin [34], was dramatically upregulated upon G9a knockdown. A recent study showed that APC and APC2 concomitantly play an important role in normal homeostasis and in tumorigenesis via suppressing Wnt signaling [18]. Interestingly, APC2 is the most frequently inactivated tumor suppressor by promoter methylation in lung cancer [19, 20]. We demonstrated that targeting G9a suppressed Wnt signaling likely through restoring Wnt inhibitors such APC2, WIF1, and DKK1 expression. This observation is further supported by the reverse correlation between G9a mRNA levels and DKK1/WIF1 revealed by mining TCGA gene expression data portal. In addition, the proto-oncogene c-Myc is also a key oncogenic component of the WNT/β-catenin signaling pathway through transcriptional repression of the secreted Wnt inhibitors DKK1 and SFRP-1 in cancer cells [49]. A recent study showed that G9a regulated breast cancer growth by repressing ferroxidase hephaestin, while G9a inhibition led to a marked down-regulation of c-Myc [36]. However, we found that knockdown of G9a did not downregulate c-Myc expression in NSCLC cells; indicating G9a may differentially regulate gene expression in different cell contexts.

Interestingly, a previous study showed the association between H3K9-me2 and aberrant Wnt/β-catenin signaling pathway in neuroendocrine tumors [24]. Other studies demonstrated that methylation of H3K9 may collaborate with DNA methylation to regulate gene expression including these Wnt antagonists in human cancer [24, 42]. And a recent study elucidated that SETDB1, another H3K9-specific histone methyltransferase, accelerated lung cancer tumorigenesis by regulating the Wnt signaling pathway [50]. Consistently, a previous study revealed that H3K9me2 was associated with aberrant Wnt/β-catenin signaling pathway in neuroendocrine tumors [24]. Consideration of HP1α’s role in gene silencing, downregulation of HP1α upon targeting G9a also contributed to restoring these tumor suppressor genes’ expression via promoter demethylation.

## Conclusions

**Fig. 7:**
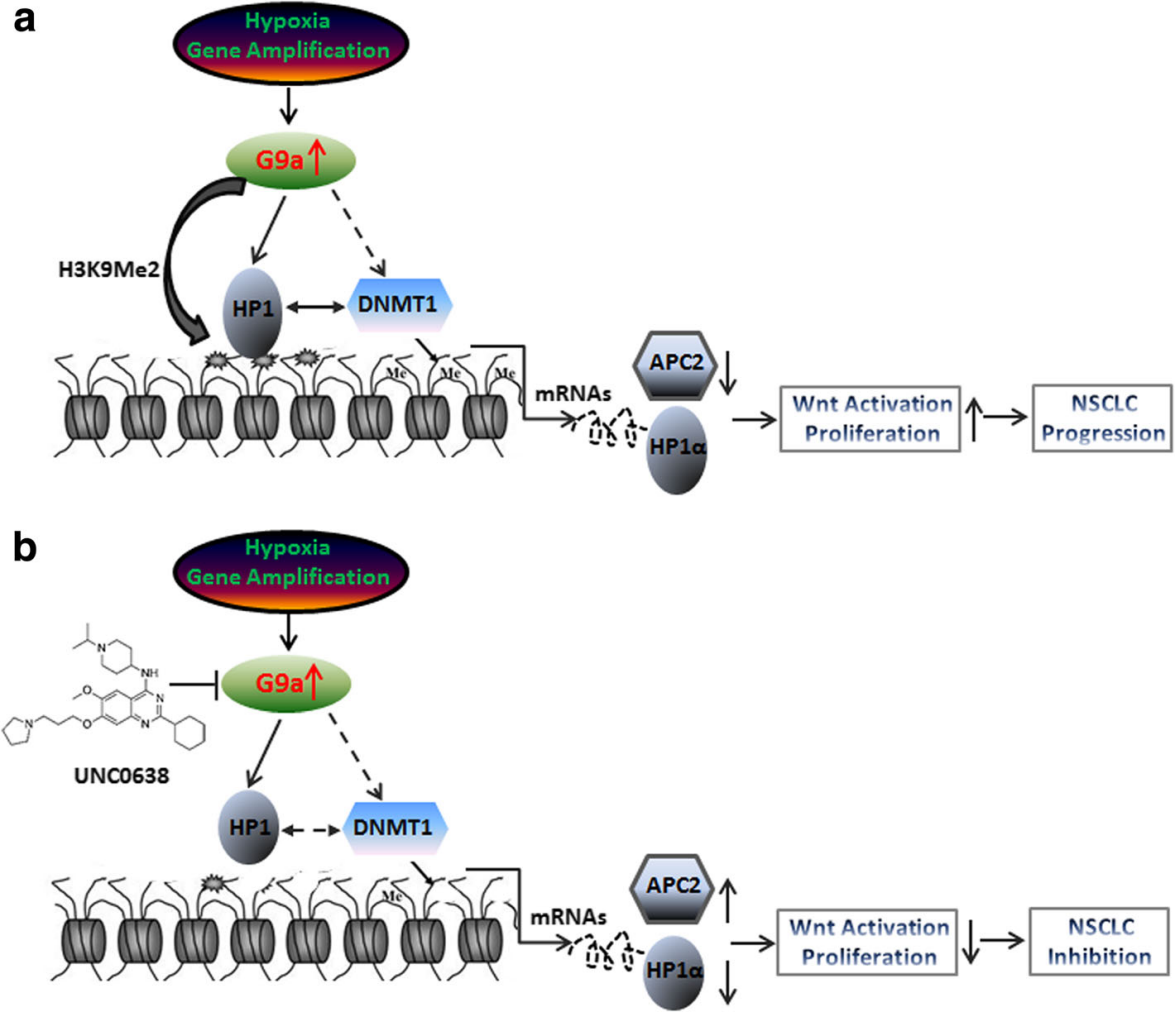
Hypothetic schematic model of the underlying mechanisms of targeting G9a to suppress NSCLC. **a** Gene amplification [14], hypoxia [51], and/or other unknown mechanisms lead G9a overexpressed in cancers. G9a dimethylates H3K9, creating a binding site for HP1 (α, β, or γ, a structural chromosomal protein involved in transcription, chromatin organization, and replication as well as DNA repair), HP1 stabilizes and enhances the DNA methyltransferase activity of DNMT1. G9a also interacts with DNMT1, and then through this interaction G9a epigenetically regulates gene expression. In lung cancer, upregulated G9a may silence gene expression of these critical inhibitors of Wnt signaling pathway such as APC2, WIF1, and other tumor suppressors through DNA hypermethylation, resulting in overactivation of Wnt signaling pathway and enhanced growth. **b** Targeting G9a by the specific inhibitor UNC0638 down-regulates HP1α, and epigenetically restores expression of APC2 and other tumor suppressors through promoter demethylation, and then significantly inhibits Wnt signaling pathways and growth of NSCLC

In summary, we found that G9a protein was overexpressed in a subgroup of NSCLC samples probably by gene amplification [14], hypoxia [51] and/or other unknown mechanisms; in collaboration with HP1α and DNMT1, G9a epigenetically regulates gene expression through H3K9-Me2 and DNA methylation. Targeting G9a significantly inhibited in vitro and in vivo growth and Wnt signaling pathways partially through down-regulating HP1α and epigenetically restoring APC2, via promoter demethylation, which finally suppressed NSCLC progression (Fig. 7). Our study suggests that G9a is a key regulator of malignancy of NSCLC, and may serve as a potential therapeutic target for NSCLC treatment.

## Additional files

Additional file 1: **Table S1.** Differentially expressed genes in three G9a-attenuated lung cancer cells (DOCX 52 kb)

Additional file 2: **Figure S1.** Inhibition of G9a induces cell cycle arrest. a A549 in G1 phase b H1299 in G2 phase. The statistical analyses of G1 and sub-G1 are shown (* *P* &lt; 0.05 compared to control siRNA). **Figure S2.** Impact of knockdown of G9a on the levels of p53 and c-Myc proteins in A549 (p53 wild-type), H1299 (p53 null), and H1975 (p53 mutated) cancer cells. **Figure S3.** Impact of knockdown of HP1α on NSCLC cells proliferation. a Western blot of HP1α in three cell lines. b Cell proliferation after 72 h posttransfection of HP1α siRNA. (*P &lt; 0.05, **P &lt; 0.01, compared to cells transfected with control siRNA. **Figure S4.** Correlation between the level of G9a mRNA with that of DKK1 mRNA and WIF1 mRNA. The statistical analysis showed that the level of G9a mRNA level is reversely correlated with that of a DKK1 mRNA (*P* = 0.00828, *R* = - 0.38), b WIF1 mRNA (*P* = 0.0403, *R* = -0.3) in 46 of LUSC tissues. X-axis is for the normalized G9a (EMHT2) mRNA level, Y-axis is for the normalized DKK1/WIF1 mRNA level. **Figure S5.** Unsupervised hierarchical clustering analysis of selected upregulated genes upon G9a knockdown. These genes include  ICAM5, JPH3, MGMT, MMP2, RASSF1α etc. that are frequently silenced by epigenetic mechanisms in lung cancers. The two top rows represent two independent siRNA repeats, Ctrl1/2 is for control siRNA and G9a-KD1/2 is for G9A siRNA1/2. **Figure S6.** UNC0638 treatment decreased HP1α in H1299 cells. Cells were treated with defined UNC0638 for 72 hours and then protein was extracted for Western blot analysis of HP1 α, G9a, H3K9-Me2. (PDF 438 kb)

## Abbreviations

ABCG2: ATP-binding cassette sub-family G member 2
ANGPTL4: Angiopoietin-like 4
APC2: The adenomatous polyposis coli-2
DKKs: DICKKOPFs
DNMT1: DNA methyltransferase 1
EHMT2: The euchromatic histone-lysine N-methyltransferase 2 gene
EMT: Epithelial-to-mesenchymal transition
EpCAM: Epithelial cellular adhesion molecule
GSEA: Gene set enrichment analysis
H3K9me1/2: Monomethylation and dimethylation of H3K9
HP1α: Heterochromatin protein 1 alpha
IC50: The half maximal inhibitory concentration
IHC: Immunohistochemistry
JPH3: Junctophilin-3
LUAD: Lung adenocarcinoma
LUSC: Lung squamous carcinoma
NSCLC: Non-small cell lung cancer
qRT-PCR: Quantitative reverse transcription polymerase chain reaction
SFRPs: Secreted Frizzled-related proteins
TCF: T-cell factor
TCGA: The Cancer Genome Atlas
TERT: Telomerase reverse transcriptase
TLN1: Talin 1
WIF1: Wnt inhibitory factor-1
