# Supplementary material for: Targeting histone methyltransferase G9a inhibits growth and Wnt signaling pathway by epigenetically regulating HP1α and APC2 gene expression in non-small cell lung cancer
Source: Mol Cancer. 2018 Oct 22;17:153. doi: 10.1186/s12943-018-0896-8 (PMC6198520; doi:10.1186/s12943-018-0896-8)
Supplement: Supplementary file 1 — Table S1. Differentially expressed genes in three G9a-attenuated lung cancer cells (DOCX 52 kb) [file 12943_2018_896_MOESM1_ESM.docx]

**Supplementary Table**

**Table S1. Differentially expressed genes in three G9a-attenuated lung cancer cells**

|  | **A549** | | **H1299** | |
| --- | --- | --- | --- | --- |
| **Gene_Symbol** | **logFC *** | **P Value** | **LogFc*** | **P Value** |
| SCG3 | 7.4415189876 | 3.11E-116 | 3.988338173 | 4.40E-113 |
| SYN1 | 5.178380814 | 1.96E-67 | 2.679579856 | 8.33E-65 |
| CPLX1 | 4.978642799 | 2.40E-79 | 4.565427995 | 1.61E-76 |
| STMN3 | 4.964740178 | 7.22E-193 | 3.335865139 | 4.60E-189 |
| RLTPR | 4.695148552 | 7.53E-58 | 1.865354905 | 2.23E-55 |
| SYP | 4.340404832 | 2.86E-124 | 3.629788653 | 6.08E-121 |
| UNC13A | 4.228239671 | 3.20E-153 | 4.610664676 | 1.36E-149 |
| BSN | 4.204800795 | 1.16E-62 | 3.409104346 | 4.09E-60 |
| FBXL16 | 4.165473006 | 8.19E-72 | 1.56467456 | 4.54E-69 |
| MYL9 | 4.01466243 | 4.84E-97 | 0.918428938 | 4.41E-94 |
| TMEM198 | 3.95778685 | 6.37E-69 | 2.758337554 | 3.12E-66 |
| MAPK8IP2 | 3.745090965 | 1.23E-100 | 2.569776431 | 1.42E-97 |
| APC2 | 3.54483975 | 7.00E-59 | 3.411206312 | 2.13E-56 |
| CAMK2N2 | 3.431589535 | 7.97E-41 | 1.40015734 | 1.20E-38 |
| CHGB | 3.360340879 | 1.15E-97 | 2.288738821 | 1.13E-94 |
| DLGAP3 | 3.343201936 | 2.39E-08 | 2.593927528 | 2.24E-07 |
| GPC2 | 3.164780647 | 1.42E-32 | 1.981259504 | 1.25E-30 |
| RAB39A | 3.102012207 | 8.93E-21 | 2.931384918 | 3.35E-19 |
| CHPF | 3.077090014 | 1.66E-94 | 2.294473957 | 1.32E-91 |
| DISP2 | 3.053991958 | 4.59E-68 | 1.545262223 | 2.09E-65 |
| PTK7 | 2.968561767 | 8.51E-50 | 1.738223457 | 1.90E-47 |
| TMEM145 | 2.967791281 | 4.30E-43 | 3.320766016 | 7.31E-41 |
| MARCH4 | 2.863826974 | 1.52E-80 | 1.173939492 | 1.08E-77 |
| CTD-3193O13.9 | 2.840157297 | 2.98E-23 | 2.050269429 | 1.40E-21 |
| EFNB3 | 2.690242586 | 4.91E-31 | 2.498943415 | 3.99E-29 |
| SPTBN4 | 2.625945684 | 1.13E-35 | 1.619716134 | 1.21E-33 |
| SNAP25 | 2.598033768 | 2.52E-86 | 2.829589972 | 1.89E-83 |
| SCN3B | 2.549596917 | 1.49E-14 | 2.367361414 | 3.15E-13 |
| PTBP2 | 2.542006929 | 2.25E-61 | 3.556432418 | 7.55E-59 |
| RTN2 | 2.540175725 | 2.05E-49 | 2.34238078 | 4.43E-47 |
| DIRAS1 | 2.536573843 | 3.88E-31 | 2.222976712 | 3.19E-29 |
| KIF5A | 2.484875768 | 5.43E-14 | 1.021326597 | 1.08E-12 |
| EMP2 | 2.460953526 | 8.98E-65 | 2.549711835 | 3.58E-62 |
| TMEM170B | 2.351859807 | 5.21E-47 | 2.819966916 | 1.01E-44 |
| CACNA1B | 2.321509325 | 0.001403557 | 3.340038203 | 0.004843297 |
| GPR137C | 2.320190465 | 9.91E-10 | 2.537190796 | 1.15E-08 |
| MAST1 | 2.302967927 | 9.11E-20 | 1.842386522 | 3.18E-18 |
| MAPK8IP1 | 2.276380907 | 3.60E-59 | 2.625946614 | 1.12E-56 |
| KIF5C | 2.254388218 | 1.81E-11 | 1.557754583 | 2.67E-10 |
| JPH3 (methylation) | 2.247281212 | 1.49E-15 | 0.951050059 | 3.46E-14 |
| FBLL1 | 2.214429189 | 0.000882861 | 1.728270397 | 0.003196202 |
| PPM1E | 2.175940611 | 1.17E-07 | 1.256664976 | 9.76E-07 |
| ACPL2 | 2.169663731 | 6.33E-39 | 2.905505177 | 8.49E-37 |
| FAM171B | 2.11717008 | 6.64E-23 | 1.639108724 | 3.01E-21 |
| THG1L | 2.101341635 | 7.47E-43 | 2.217720595 | 1.21E-40 |
| NOVA2 | 2.100040612 | 2.34E-19 | 1.192915761 | 7.90E-18 |
| NID1 metastasis | 2.075987787 | 3.78E-34 | 2.126273251 | 3.73E-32 |
| PALD1 | 2.054597636 | 2.38E-39 | 1.272661073 | 3.26E-37 |
| GOLGA7B | 2.008372633 | 2.79E-33 | 2.321325667 | 2.62E-31 |
| SLC41A1 | 2.000823727 | 4.67E-47 | 2.282840258 | 9.16E-45 |
| MEGF6 | 1.984838993 | 1.31E-17 | 1.150602479 | 3.76E-16 |
| ASPHD2 | 1.972437184 | 2.81E-15 | 1.559435955 | 6.36E-14 |
| TERT | 1.953613581 | 5.03E-12 | 2.570210921 | 7.86E-11 |
| C3orf80 | 1.951817612 | 1.23E-19 | 1.427052846 | 4.25E-18 |
| SBK1 | 1.926771218 | 2.53E-23 | 1.143493057 | 1.20E-21 |
| NUDT15 | 1.923947247 | 2.43E-31 | 2.009951501 | 2.04E-29 |
| YWHAH | 1.922926341 | 2.65E-49 | 2.018435139 | 5.63E-47 |
| CCDC64 | 1.889222846 | 2.17E-26 | 2.564927992 | 1.31E-24 |
| RIPPLY2 | 1.884072341 | 0.00017409 | 1.262949891 | 0.000749705 |
| WNT5B | 1.882405131 | 1.34E-06 | 2.881836977 | 9.25E-06 |
| STEAP3 | 1.855498422 | 2.76E-45 | 2.255251155 | 5.02E-43 |
| KCNG3 | 1.855020611 | 0.000771467 | 0.94180155 | 0.002843808 |
| ADC | 1.851693967 | 3.21E-18 | 1.71725166 | 9.66E-17 |
| LRRC24 | 1.832661326 | 4.01E-19 | 1.350464749 | 1.31E-17 |
| SLC6A17 | 1.826590757 | 3.51E-15 | 1.107720187 | 7.86E-14 |
| CACNB4 | 1.821853427 | 0.000356816 | 1.372157202 | 0.001430745 |
| KDELC2 | 1.800487191 | 6.50E-28 | 1.223798446 | 4.39E-26 |
| PODXL2 | 1.796696585 | 5.34E-36 | 2.62628129 | 5.92E-34 |
| SS18L1 | 1.782412668 | 3.65E-24 | 1.664435629 | 1.87E-22 |
| GPR153 | 1.770243724 | 2.06E-24 | 3.274558187 | 1.08E-22 |
| ATP1A3 | 1.767132555 | 8.66E-17 | 2.250412676 | 2.27E-15 |
| PPFIA3 | 1.766256303 | 3.25E-30 | 1.032836129 | 2.51E-28 |
| TLN1 (promote?) | 1.739585381 | 3.47E-31 | 2.178947891 | 2.89E-29 |
| SARM1 | 1.723385449 | 6.83E-36 | 0.953539023 | 7.51E-34 |
| CYB5R4 | 1.712562499 | 7.15E-33 | 2.146676669 | 6.51E-31 |
| IL6ST | 1.698909502 | 6.87E-13 | 1.982978491 | 1.20E-11 |
| MAPK11 | 1.694689536 | 1.60E-19 | 1.615037511 | 5.45E-18 |
| MTURN | 1.686031313 | 1.32E-23 | 2.88105531 | 6.38E-22 |
| RBM38 | 1.683396116 | 3.44E-33 | 2.240797153 | 3.20E-31 |
| GPR161 | 1.67461485 | 3.31E-28 | 1.298669499 | 2.28E-26 |
| TBX1 | 1.643471841 | 2.85E-07 | 1.275565922 | 2.23E-06 |
| HCN3 | 1.634445251 | 9.08E-12 | 1.77742538 | 1.38E-10 |
| SCAMP5 | 1.634211608 | 1.13E-32 | 1.10444039 | 1.01E-30 |
| MAATS1 | 1.616094997 | 0.000549536 | 1.13381795 | 0.002114378 |
| CBS | 1.601478197 | 7.75E-28 | 1.161025772 | 5.17E-26 |
| MFAP5 | 1.601216476 | 0.002792394 | 1.838026605 | 0.008874256 |
| ELOVL7 | 1.586944578 | 3.55E-16 | 2.80035135 | 8.74E-15 |
| RAB15 | 1.551012825 | 5.01E-34 | 1.995208626 | 4.92E-32 |
| HS6ST1 | 1.55003894 | 5.96E-27 | 2.355819335 | 3.76E-25 |
| POLR3E | 1.547649106 | 1.38E-30 | 1.502733355 | 1.08E-28 |
| PHF1 | 1.541517029 | 8.32E-24 | 0.90122621 | 4.10E-22 |
| FBXO41 | 1.535170288 | 1.67E-21 | 0.939284256 | 6.71E-20 |
| RIMS2 | 1.532649109 | 5.52E-08 | 1.402473752 | 4.86E-07 |
| ALCAM | 1.524614188 | 6.22E-23 | 2.040163134 | 2.84E-21 |
| TMEM184B | 1.510002905 | 3.83E-35 | 1.695069414 | 4.06E-33 |
| SFT2D3 | 1.504476454 | 1.73E-17 | 1.333040544 | 4.87E-16 |
| MTPN | 1.501960544 | 1.67E-22 | 0.970128058 | 7.25E-21 |
| GOLGA4 | 1.497843508 | 3.26E-23 | 2.017629019 | 1.51E-21 |
| GAS6 | 1.494920489 | 4.18E-25 | 1.354049011 | 2.30E-23 |
| ZYX | 1.488552733 | 3.29E-29 | 1.282448121 | 2.37E-27 |
| CLCN6 | 1.473278885 | 2.49E-22 | 1.609663678 | 1.07E-20 |
| FAM221A | 1.471334204 | 3.90E-05 | 1.071338913 | 0.000195994 |
| GABBR1 | 1.462391181 | 1.07E-08 | 1.059023214 | 1.06E-07 |
| RBM15B | 1.459730589 | 7.85E-33 | 1.437220338 | 7.04E-31 |
| BDNF | 1.45737253 | 2.08E-29 | 3.45399914 | 1.52E-27 |
| ZFYVE9 | 1.456220002 | 8.60E-23 | 2.060368271 | 3.82E-21 |
| STX2 | 1.455979189 | 1.38E-27 | 1.379202335 | 9.02E-26 |
| PAQR4 | 1.450197279 | 1.09E-27 | 2.101305161 | 7.23E-26 |
| GNG4 | 1.443368801 | 1.01E-22 | 3.211529063 | 4.48E-21 |
| DNMT1 | 1.438917444 | 6.80E-26 | 1.800870745 | 3.94E-24 |
| SBNO1 | 1.436948751 | 2.09E-24 | 1.669117627 | 1.10E-22 |
| KLRG1 | 1.436190665 | 2.80E-12 | 1.761275551 | 4.58E-11 |
| HCN2 | 1.435045509 | 3.33E-23 | 2.456395709 | 1.54E-21 |
| ZNF296 | 1.426100001 | 7.47E-09 | 1.041515923 | 7.56E-08 |
| ANKRD13B | 1.42403474 | 7.66E-24 | 2.172266505 | 3.78E-22 |
| ADAM11 | 1.423217458 | 9.44E-07 | 1.484226272 | 6.73E-06 |
| UCK1 | 1.41200738 | 9.88E-22 | 2.037964994 | 4.05E-20 |
| WDR65 | 1.410100897 | 0.000310132 | 1.421382711 | 0.001259799 |
| ZNF512 | 1.397992394 | 9.59E-28 | 1.264644292 | 6.37E-26 |
| SMAD2 TGFB | 1.390565095 | 5.94E-27 | 1.004845374 | 3.76E-25 |
| CORO6 | 1.388370351 | 1.53E-05 | 1.135299048 | 8.45E-05 |
| QKI | 1.369279306 | 6.66E-17 | 2.109237581 | 1.76E-15 |
| RAB3B | 1.368231453 | 9.37E-15 | 1.759025775 | 2.01E-13 |
| MEX3D | 1.36425753 | 1.73E-22 | 1.133598418 | 7.48E-21 |
| ICAM5 | 1.361537763 | 0.000314746 | 1.462083819 | 0.001276915 |
| EID3 | 1.359648341 | 7.40E-09 | 1.466560235 | 7.50E-08 |
| FHAD1 | 1.358927435 | 0.000746451 | 1.534286357 | 0.002763582 |
| ZDHHC20 | 1.353389176 | 1.31E-06 | 1.876267102 | 9.05E-06 |
| CADM4 | 1.349496932 | 2.36E-13 | 1.695802574 | 4.34E-12 |
| PIGW | 1.342440314 | 1.05E-21 | 1.5618167 | 4.31E-20 |
| CCDC136 | 1.340687186 | 8.19E-05 | 1.125922394 | 0.000384755 |
| ZSWIM8 | 1.328487248 | 4.04E-24 | 1.610357608 | 2.05E-22 |
| EML5 | 1.32759372 | 3.30E-08 | 1.317052224 | 3.03E-07 |
| PRRC2B | 1.326875414 | 2.08E-17 | 1.679633446 | 5.84E-16 |
| AKAP8 | 1.321093105 | 2.25E-21 | 1.266979601 | 8.92E-20 |
| RIMS3 | 1.31985276 | 2.07E-10 | 1.509409296 | 2.65E-09 |
| DENND6B | 1.316023327 | 2.96E-08 | 1.271678029 | 2.74E-07 |
| TCAP | 1.314630717 | 5.43E-06 | 1.933182621 | 3.32E-05 |
| KIRREL2 | 1.314288916 | 6.11E-09 | 1.245736912 | 6.29E-08 |
| POLD3 | 1.312206201 | 3.64E-22 | 1.762847832 | 1.56E-20 |
| HOXB9 | 1.300148886 | 4.26E-21 | 1.055380677 | 1.65E-19 |
| CMTM1 | 1.29661448 | 2.24E-07 | 1.395976765 | 1.78E-06 |
| MMP24 | 1.291324315 | 1.63E-23 | 4.370790514 | 7.83E-22 |
| FAM102B | 1.290541992 | 1.15E-13 | 1.998704757 | 2.21E-12 |
| RAB36 | 1.2872979 | 1.23E-15 | 0.98794311 | 2.89E-14 |
| BIVM | 1.284973441 | 5.88E-18 | 0.909975971 | 1.73E-16 |
| SNRNP27 | 1.284215946 | 8.11E-17 | 1.418083889 | 2.13E-15 |
| SHANK3 | 1.278468253 | 1.03E-08 | 1.887083471 | 1.02E-07 |
| PTBP3 | 1.27822624 | 2.05E-14 | 2.298211017 | 4.26E-13 |
| NLGN2 | 1.276382529 | 1.46E-20 | 1.635842467 | 5.42E-19 |
| CNNM4 | 1.276300883 | 3.06E-17 | 1.488137263 | 8.42E-16 |
| ATL1 | 1.271583788 | 2.25E-07 | 1.051263101 | 1.79E-06 |
| HOMER1 | 1.27018306 | 2.74E-13 | 1.760279717 | 4.99E-12 |
| DXO | 1.267934463 | 4.31E-11 | 1.169526173 | 6.03E-10 |
| GYLTL1B | 1.266103987 | 9.63E-09 | 2.545926042 | 9.60E-08 |
| HERPUD2 | 1.265758191 | 8.85E-21 | 1.288864822 | 3.34E-19 |
| PODXL | 1.261289528 | 3.24E-20 | 1.662129749 | 1.17E-18 |
| WHAMM | 1.256706927 | 1.90E-16 | 0.963225798 | 4.78E-15 |
| DHX40 | 1.249230623 | 1.13E-22 | 2.051375843 | 5.00E-21 |
| TCFL5 | 1.244366429 | 1.52E-16 | 1.286729085 | 3.88E-15 |
| DFFB | 1.236953589 | 9.24E-12 | 1.38060935 | 1.40E-10 |
| NSUN7 | 1.235300912 | 0.000245834 | 0.949588599 | 0.001024068 |
| RRAGB | 1.232484527 | 1.71E-17 | 1.006508943 | 4.84E-16 |
| TMEM41A | 1.228417361 | 2.40E-20 | 1.050443926 | 8.76E-19 |
| TTC13 | 1.2271716 | 2.70E-21 | 1.113702373 | 1.06E-19 |
| ADAMTSL4 | 1.22628404 | 1.84E-09 | 1.684604605 | 2.06E-08 |
| ITPKA | 1.212310629 | 6.28E-05 | 0.943641545 | 0.000302105 |
| LAMC3 | 1.208227953 | 0.001001346 | 1.444686403 | 0.0035774 |
| PIGZ | 1.203752746 | 3.20E-07 | 1.76466437 | 2.49E-06 |
| MFSD6 | 1.199552769 | 4.40E-12 | 1.619890923 | 6.92E-11 |
| DCLK2 | 1.184176322 | 1.89E-14 | 1.220788295 | 3.94E-13 |
| CAMLG | 1.183889458 | 6.64E-19 | 1.345975344 | 2.11E-17 |
| BET1 | 1.163301985 | 5.63E-15 | 1.094223131 | 1.24E-13 |
| ATF2 | 1.156327956 | 6.48E-16 | 2.139983313 | 1.56E-14 |
| CALML4 | 1.147157568 | 1.71E-06 | 1.653632474 | 1.16E-05 |
| MOGS | 1.144682391 | 1.18E-19 | 1.862799332 | 4.09E-18 |
| SGTA | 1.143719579 | 7.76E-16 | 1.292842418 | 1.85E-14 |
| THUMPD1 | 1.143171651 | 2.23E-17 | 1.144795555 | 6.24E-16 |
| MDGA1 | 1.132474496 | 1.48E-11 | 1.267316218 | 2.21E-10 |
| AUNIP | 1.12018056 | 1.32E-08 | 2.194022751 | 1.28E-07 |
| SEC24B | 1.11206805 | 3.52E-12 | 1.709293451 | 5.67E-11 |
| RADIL | 1.1120408 | 3.81E-06 | 1.163379091 | 2.41E-05 |
| MDK | 1.11098552 | 4.32E-13 | 1.691677662 | 7.72E-12 |
| YES1 drug resistance | 1.102710611 | 1.49E-13 | 1.044896874 | 2.81E-12 |
| CNOT3 | 1.091555224 | 2.65E-16 | 1.816724498 | 6.57E-15 |
| AIF1L | 1.089036483 | 0.003641139 | 1.992952923 | 0.011178612 |
| RP11-111M22.2 | 1.085673886 | 0.000454975 | 0.971158676 | 0.001779554 |
| DPYSL5 | 1.084070819 | 2.61E-10 | 1.245394619 | 3.30E-09 |
| PASK | 1.083286453 | 1.41E-09 | 1.524251573 | 1.60E-08 |
| PGRMC2 | 1.082338165 | 2.48E-16 | 2.2020702 | 6.16E-15 |
| KIAA0100 | 1.080654488 | 1.54E-11 | 1.765635164 | 2.28E-10 |
| GBX2 | 1.080188314 | 0.000302597 | 1.3707143 | 0.001232333 |
| C18orf54 | 1.079727787 | 1.21E-09 | 1.081428609 | 1.39E-08 |
| SOCS4 | 1.079262201 | 1.11E-07 | 1.101716532 | 9.28E-07 |
| TMEM201 | 1.078054717 | 5.28E-14 | 1.47280054 | 1.05E-12 |
| LSM7 | 1.072087223 | 1.67E-09 | 1.339294976 | 1.87E-08 |
| PACS2 | 1.071472306 | 1.25E-16 | 1.337858514 | 3.22E-15 |
| RAB11FIP4 | 1.070227748 | 1.03E-09 | 2.428065537 | 1.19E-08 |
| RASGEF1A | 1.065564978 | 9.42E-08 | 1.008783818 | 7.96E-07 |
| LRFN1 | 1.062818692 | 1.35E-12 | 1.149123684 | 2.29E-11 |
| COL11A1 | 1.061995963 | 8.95E-13 | 1.176755032 | 1.54E-11 |
| STARD9 | 1.06050278 | 0.001650104 | 1.180760859 | 0.005586686 |
| NT5DC2 | 1.058765979 | 7.89E-14 | 2.399827113 | 1.54E-12 |
| ASIC1 | 1.058398948 | 2.44E-13 | 1.057070743 | 4.47E-12 |
| FBLN2 | 1.057855195 | 0.001260086 | 1.825178881 | 0.004393414 |
| LIMCH1 | 1.057771488 | 1.33E-14 | 1.328755657 | 2.84E-13 |
| FKBP11 | 1.056635249 | 4.18E-10 | 1.20527546 | 5.15E-09 |
| NDUFA5 | 1.054195929 | 1.98E-12 | 1.430585071 | 3.29E-11 |
| SAMD5 | 1.049980452 | 5.01E-07 | 0.950393215 | 3.77E-06 |
| E2F2 | 1.049534804 | 3.84E-11 | 1.299734348 | 5.41E-10 |
| FBXO48 | 1.049518487 | 3.09E-06 | 1.097718537 | 2.00E-05 |
| CIPC | 1.046563974 | 3.28E-12 | 1.669357702 | 5.29E-11 |
| CELSR2 | 1.043874929 | 1.52E-11 | 3.745330519 | 2.26E-10 |
| RTN4R | 1.042379279 | 2.32E-06 | 1.024366724 | 1.53E-05 |
| TGFB2 | 1.040572578 | 1.47E-14 | 3.207821033 | 3.12E-13 |
| TMEM50B | 1.039083255 | 5.34E-13 | 1.649245235 | 9.47E-12 |
| TMEM180 | 1.037454736 | 1.30E-11 | 0.98666388 | 1.95E-10 |
| GARNL3 | 1.03727159 | 0.019947468 | 1.416643311 | 0.0477862 |
| SS18 | 1.036280457 | 9.57E-16 | 1.330287575 | 2.26E-14 |
| MED15 | 1.032265847 | 9.36E-14 | 1.393110399 | 1.82E-12 |
| CDK14 | 1.032145262 | 4.14E-06 | 1.655987124 | 2.60E-05 |
| SPOPL | 1.028146841 | 6.20E-09 | 1.689408316 | 6.37E-08 |
| KCTD14 | 1.019826771 | 0.002085613 | 1.375958097 | 0.006862343 |
| PPP1R12B | 1.016656394 | 3.14E-08 | 1.990889289 | 2.89E-07 |
| LRRC8C | 1.016493511 | 3.84E-09 | 1.110283026 | 4.10E-08 |
| RPGRIP1L | 1.016279886 | 2.32E-12 | 1.679771065 | 3.83E-11 |
| SRRM3 | 1.011647676 | 9.60E-09 | 2.589110979 | 9.58E-08 |
| PYGO1 | 1.011250609 | 8.52E-07 | 1.417866651 | 6.12E-06 |
| UHMK1 | 1.007755213 | 5.49E-06 | 1.550994961 | 3.35E-05 |
| GZF1 | 1.004939761 | 2.72E-14 | 1.023114539 | 5.61E-13 |
| IPO11 | 1.004601498 | 2.11E-12 | 1.655954721 | 3.50E-11 |
| PALM | 1.00069359 | 2.42E-11 | 2.01111319 | 3.51E-10 |
| BAK1 | -1.000061926 | 3.82E-12 | -1.26978936 | 6.10E-11 |
| GPC6 | -1.002157052 | 6.94E-08 | -1.151145251 | 5.98E-07 |
| BVES | -1.003969704 | 4.46E-09 | -1.224623153 | 4.71E-08 |
| BAIAP3 | -1.008896675 | 0.000816551 | -1.893450602 | 0.002990111 |
| ELL2 | -1.010330213 | 2.01E-15 | -0.934777641 | 4.60E-14 |
| TOR2A | -1.011487888 | 7.58E-10 | -1.441111572 | 8.98E-09 |
| PFN4 | -1.013085761 | 0.003998139 | -1.907500941 | 0.012157509 |
| TRIML2 | -1.014990899 | 1.66E-10 | -2.777066062 | 2.17E-09 |
| DDTL | -1.021457846 | 0.004199332 | -1.059584191 | 0.012693594 |
| BIK | -1.023334446 | 0.000307767 | -1.383659542 | 0.001251389 |
| ZFP69B | -1.024306171 | 0.002385154 | -1.455164517 | 0.007718597 |
| RARRES3 | -1.027322226 | 1.60E-05 | -1.655005643 | 8.76E-05 |
| CTGF | -1.030126834 | 2.36E-16 | -1.866070321 | 5.89E-15 |
| SF3B14 | -1.034448508 | 1.09E-10 | -1.496687727 | 1.46E-09 |
| OSGIN1 | -1.035731058 | 8.67E-12 | -2.783930969 | 1.32E-10 |
| F2RL2 | -1.039451947 | 1.84E-11 | -4.807591371 | 2.71E-10 |
| BLOC1S5 | -1.046409072 | 2.26E-11 | -1.088904534 | 3.29E-10 |
| TMF1 | -1.046534217 | 3.04E-09 | -1.685681247 | 3.30E-08 |
| SYNPO2 | -1.048103287 | 1.31E-07 | -1.029512131 | 1.08E-06 |
| PBXIP1 | -1.048632031 | 6.31E-16 | -1.292872835 | 1.53E-14 |
| WDR7 | -1.049606144 | 3.39E-07 | -1.035999972 | 2.62E-06 |
| TLR6 | -1.051454064 | 3.54E-07 | -1.348165964 | 2.74E-06 |
| PAFAH2 | -1.057392035 | 2.82E-16 | -1.575233074 | 6.99E-15 |
| ATP8B2 | -1.057668097 | 2.99E-13 | -1.009541755 | 5.43E-12 |
| EMC7 | -1.059215497 | 6.59E-15 | -0.984170666 | 1.44E-13 |
| DCP1A | -1.060119807 | 2.86E-15 | -1.07984947 | 6.45E-14 |
| PTAR1 | -1.060758108 | 2.65E-05 | -1.286421635 | 0.000137359 |
| C7orf43 | -1.063288911 | 9.21E-13 | -1.163332598 | 1.58E-11 |
| KCNF1 | -1.063922892 | 2.09E-09 | -6.047830097 | 2.32E-08 |
| CCDC68 | -1.065475301 | 2.16E-09 | -2.252518889 | 2.39E-08 |
| C5orf55 | -1.067245502 | 2.42E-06 | -0.928018517 | 1.59E-05 |
| CD46 | -1.067675885 | 5.99E-16 | -1.34338487 | 1.46E-14 |
| ZDHHC21 | -1.069387539 | 3.83E-09 | -1.413599323 | 4.10E-08 |
| TUBA1A | -1.072739676 | 4.64E-16 | -1.070519622 | 1.14E-14 |
| NDRG4 | -1.076351356 | 4.32E-17 | -1.063242248 | 1.17E-15 |
| BLOC1S6 | -1.08169385 | 3.14E-15 | -1.649732423 | 7.06E-14 |
| MAP2K1 | -1.082379739 | 2.18E-18 | -1.256922651 | 6.63E-17 |
| CFD | -1.082484915 | 4.98E-08 | -1.254621401 | 4.42E-07 |
| RNF125 | -1.082735453 | 3.69E-05 | -1.05665313 | 0.000185827 |
| C11orf45 | -1.08529275 | 3.14E-07 | -1.242576753 | 2.45E-06 |
| HKDC1 | -1.086973225 | 1.72E-16 | -2.679275879 | 4.35E-15 |
| SETD8 | -1.094093525 | 1.88E-18 | -0.97409471 | 5.76E-17 |
| ETV1 | -1.095159362 | 2.00E-13 | -1.042961827 | 3.72E-12 |
| ACSL5 | -1.09521356 | 6.11E-08 | -1.010860703 | 5.33E-07 |
| NAB1 | -1.095270321 | 1.30E-11 | -1.758234238 | 1.95E-10 |
| FAM214A | -1.095855118 | 2.98E-11 | -1.12901511 | 4.27E-10 |
| TMBIM1 | -1.095892938 | 2.96E-18 | -1.808102432 | 8.94E-17 |
| KIAA1217 | -1.096710305 | 8.37E-12 | -0.954331202 | 1.28E-10 |
| FLRT3 | -1.09900938 | 1.11E-15 | -1.629711526 | 2.62E-14 |
| IGF2R | -1.10040234 | 1.65E-08 | -1.106481816 | 1.58E-07 |
| POLR3GL | -1.100842693 | 4.96E-09 | -1.351980281 | 5.20E-08 |
| DNAJA2 | -1.101293611 | 2.09E-19 | -1.226847519 | 7.07E-18 |
| GJB2 | -1.103536554 | 1.20E-06 | -1.392736387 | 8.34E-06 |
| OVGP1 | -1.103645765 | 5.96E-05 | -1.224608107 | 0.00028784 |
| PPM1K | -1.104582498 | 1.33E-11 | -1.596474381 | 1.99E-10 |
| DDX60L | -1.105136193 | 3.88E-12 | -1.703711333 | 6.20E-11 |
| DPH6 | -1.106020955 | 3.82E-09 | -1.898301514 | 4.10E-08 |
| LAT2 | -1.108682965 | 0.000116637 | -2.679871547 | 0.000525732 |
| FYTTD1 | -1.115090428 | 5.69E-16 | -0.974207495 | 1.39E-14 |
| REST | -1.116254141 | 2.38E-12 | -1.71713034 | 3.91E-11 |
| TLL1 | -1.121772519 | 6.68E-06 | -1.696191542 | 4.00E-05 |
| WBSCR27 | -1.122392546 | 5.18E-10 | -1.862871717 | 6.31E-09 |
| HIST1H3H | -1.127932414 | 0.001003137 | -2.331971577 | 0.003582791 |
| SLC25A35 | -1.129162633 | 2.87E-09 | -1.953882505 | 3.13E-08 |
| POT1 | -1.131537166 | 3.49E-17 | -1.493635424 | 9.55E-16 |
| TSPAN1 | -1.132426469 | 0.000301569 | -2.382120421 | 0.001229719 |
| ACOT4 | -1.134224376 | 4.00E-07 | -2.564440422 | 3.05E-06 |
| IGIP | -1.138401766 | 1.44E-08 | -1.911696506 | 1.39E-07 |
| SFXN3 | -1.140379162 | 4.24E-17 | -2.422374555 | 1.15E-15 |
| DPY30 | -1.140923781 | 1.63E-16 | -2.097533021 | 4.13E-15 |
| ARL15 | -1.142116735 | 9.07E-12 | -1.529121309 | 1.38E-10 |
| KLHL15 | -1.142218162 | 2.99E-08 | -1.052442124 | 2.76E-07 |
| KIDINS220 | -1.14474728 | 3.18E-09 | -1.172013575 | 3.44E-08 |
| MAST4 | -1.14798657 | 1.87E-11 | -2.459487786 | 2.75E-10 |
| SCCPDH | -1.150227923 | 6.97E-19 | -1.336413646 | 2.20E-17 |
| METAP1 | -1.156550067 | 7.66E-20 | -1.762669612 | 2.71E-18 |
| LIPH | -1.157274906 | 9.73E-19 | -5.4895559 | 3.03E-17 |
| ELOVL6 | -1.160916903 | 1.47E-18 | -1.176668739 | 4.51E-17 |
| HMGCL | -1.161829647 | 2.44E-13 | -1.910963474 | 4.47E-12 |
| ARHGAP18 | -1.164632031 | 3.00E-17 | -2.053820063 | 8.28E-16 |
| UNC45A | -1.174020018 | 3.80E-20 | -1.038587358 | 1.37E-18 |
| LMBRD1 | -1.174690226 | 3.91E-19 | -2.013145228 | 1.28E-17 |
| TIMM17A | -1.179473933 | 4.92E-17 | -1.714097931 | 1.32E-15 |
| TSNAX | -1.181337474 | 3.67E-14 | -1.854556334 | 7.44E-13 |
| CPEB2 | -1.191730193 | 5.58E-09 | -1.23764574 | 5.78E-08 |
| RDH10 | -1.19382182 | 1.41E-21 | -1.240426275 | 5.75E-20 |
| ADAMTS16 | -1.195584382 | 1.45E-08 | -2.880905929 | 1.40E-07 |
| MAPK8 | -1.197125817 | 4.34E-17 | -1.085094748 | 1.17E-15 |
| GEM | -1.198442421 | 9.30E-16 | -1.818423826 | 2.21E-14 |
| CARD6 | -1.199174719 | 2.63E-14 | -1.406091892 | 5.43E-13 |
| MBLAC2 | -1.200173655 | 5.64E-10 | -1.361123181 | 6.81E-09 |
| PCDH18 | -1.201014948 | 0.001404196 | -1.100667417 | 0.004844191 |
| NXPE3 | -1.20413303 | 6.17E-16 | -2.112643069 | 1.50E-14 |
| SMIM8 | -1.207678912 | 4.38E-06 | -1.135145473 | 2.73E-05 |
| AQP3 | -1.208107974 | 4.73E-12 | -2.196492406 | 7.41E-11 |
| SDSL | -1.208214012 | 1.38E-15 | -1.897061496 | 3.22E-14 |
| GIPR | -1.211952388 | 0.000137857 | -2.680571158 | 0.000610586 |
| MOSPD2 | -1.21350511 | 6.11E-15 | -1.593288295 | 1.34E-13 |
| GALNT4 | -1.216223083 | 7.44E-05 | -3.498331135 | 0.000352217 |
| MAP2K4 | -1.219039892 | 8.51E-19 | -1.7776488 | 2.66E-17 |
| SNRK | -1.220539003 | 5.37E-10 | -1.535065767 | 6.53E-09 |
| SMPD1 | -1.221884881 | 3.47E-19 | -1.03672669 | 1.14E-17 |
| ECHDC1 | -1.226926505 | 8.41E-20 | -1.206371243 | 2.96E-18 |
| TCEA3 | -1.228855435 | 0.001035507 | -0.958974264 | 0.003680822 |
| NECAP1 | -1.229317031 | 1.98E-19 | -1.214384697 | 6.76E-18 |
| RHOU | -1.231034323 | 4.25E-18 | -2.293587207 | 1.27E-16 |
| CXADR | -1.231705171 | 6.49E-17 | -0.987357764 | 1.72E-15 |
| CREBL2 | -1.234261584 | 5.44E-22 | -1.862319364 | 2.29E-20 |
| GUCY1B3 | -1.234382908 | 6.16E-09 | -0.995537587 | 6.33E-08 |
| ANGPTL4 | -1.23491466 | 7.94E-09 | -3.818963979 | 8.01E-08 |
| CPE | -1.236362804 | 2.03E-19 | -1.191508905 | 6.89E-18 |
| ANK3 | -1.236393399 | 3.32E-10 | -2.180390327 | 4.13E-09 |
| ANAPC13 | -1.239585369 | 4.41E-18 | -1.495029449 | 1.31E-16 |
| PTPRM | -1.240515483 | 1.41E-19 | -1.132046231 | 4.83E-18 |
| PAXIP1-AS2 | -1.247167476 | 2.51E-06 | -1.123311389 | 1.64E-05 |
| BMP2K | -1.24824108 | 1.11E-11 | -1.251118116 | 1.68E-10 |
| HNRNPH2 | -1.250391654 | 1.66E-23 | -1.819235286 | 7.94E-22 |
| PCDHGB3 | -1.250435302 | 0.002563316 | -1.114379823 | 0.008226232 |
| ZNF470 | -1.252486112 | 2.21E-07 | -1.005127047 | 1.76E-06 |
| C15orf57 | -1.253009725 | 9.15E-16 | -1.431906343 | 2.18E-14 |
| SERTAD2 | -1.256037286 | 4.38E-15 | -1.117159221 | 9.75E-14 |
| CHIC1 | -1.25750602 | 3.02E-10 | -1.537773328 | 3.79E-09 |
| WDFY2 | -1.257947003 | 1.79E-14 | -1.329915488 | 3.77E-13 |
| RP11-181C3.1 | -1.258358831 | 0.001149143 | -1.569788931 | 0.004047561 |
| TAPBPL | -1.262143165 | 9.08E-12 | -1.883903082 | 1.38E-10 |
| EPM2A | -1.263364272 | 4.64E-11 | -1.272824786 | 6.45E-10 |
| SCNN1A | -1.263412429 | 8.29E-09 | -1.66219026 | 8.34E-08 |
| SLC10A7 | -1.264162447 | 1.84E-08 | -1.09468331 | 1.75E-07 |
| LHX1 | -1.264394734 | 1.45E-09 | -2.586788421 | 1.65E-08 |
| DNAJC13 | -1.264965352 | 8.94E-14 | -1.365855574 | 1.74E-12 |
| MAP4K5 | -1.26668372 | 4.82E-19 | -1.695152837 | 1.56E-17 |
| ZNF461 | -1.270424137 | 3.18E-10 | -1.029707504 | 3.97E-09 |
| NUDT9 | -1.27068064 | 1.95E-21 | -2.068115233 | 7.78E-20 |
| MAPK10 | -1.274960267 | 1.07E-06 | -3.067897038 | 7.60E-06 |
| MICU3 | -1.276355391 | 7.46E-11 | -1.36106638 | 1.02E-09 |
| CNTN1 | -1.276676264 | 7.49E-11 | -1.506674862 | 1.02E-09 |
| SNCG | -1.276715801 | 3.00E-07 | -3.738266474 | 2.34E-06 |
| MYLIP | -1.280382148 | 1.80E-14 | -0.964480491 | 3.78E-13 |
| TMEM183A | -1.281252447 | 1.58E-21 | -2.010120015 | 6.36E-20 |
| LAMA2 | -1.281874256 | 0.0001746 | -1.78379703 | 0.000751393 |
| TM4SF18 | -1.284122052 | 9.36E-22 | -1.821529521 | 3.85E-20 |
| GLYCTK | -1.286139229 | 1.00E-08 | -2.192207759 | 9.95E-08 |
| SLC9A6 | -1.289980919 | 1.11E-16 | -0.972242593 | 2.88E-15 |
| ZNF138 | -1.292886217 | 4.07E-13 | -0.966218799 | 7.32E-12 |
| THRB | -1.293849849 | 1.49E-16 | -1.486588077 | 3.81E-15 |
| IL31RA | -1.296465216 | 2.47E-07 | -0.976915342 | 1.95E-06 |
| KLHL28 | -1.297335729 | 8.75E-09 | -1.569484302 | 8.80E-08 |
| ZSCAN18 | -1.307918853 | 7.63E-13 | -1.241288111 | 1.33E-11 |
| HBEGF | -1.308010385 | 5.40E-10 | -1.982374678 | 6.56E-09 |
| LDHD | -1.310555921 | 4.84E-07 | -1.951407246 | 3.65E-06 |
| POR | -1.310926112 | 1.57E-23 | -1.76449292 | 7.53E-22 |
| B3GNT3 | -1.312643183 | 2.23E-17 | -1.375551293 | 6.24E-16 |
| FUOM | -1.312850771 | 6.62E-08 | -1.113479144 | 5.74E-07 |
| CLU | -1.314252283 | 4.47E-20 | -1.831168182 | 1.61E-18 |
| TMEM27 | -1.3224797 | 1.48E-07 | -1.203785716 | 1.21E-06 |
| GBP3 | -1.323884848 | 1.57E-24 | -1.791707744 | 8.39E-23 |
| NHLRC3 | -1.323974044 | 1.65E-17 | -1.853345724 | 4.70E-16 |
| SCML1 | -1.325151073 | 1.74E-16 | -1.244535325 | 4.40E-15 |
| RAD17 | -1.325400682 | 3.68E-23 | -2.163749501 | 1.69E-21 |
| SGPP1 | -1.325411294 | 1.84E-21 | -1.342109556 | 7.35E-20 |
| SYPL1 | -1.32584854 | 1.31E-26 | -1.494907575 | 8.04E-25 |
| UNC13D | -1.32626488 | 2.74E-06 | -1.240975219 | 1.78E-05 |
| NRAS | -1.326888265 | 5.36E-21 | -1.24390281 | 2.05E-19 |
| ABCG2 | -1.328009404 | 2.84E-15 | -2.053255629 | 6.43E-14 |
| AMMECR1 | -1.330743413 | 5.14E-22 | -1.19178636 | 2.18E-20 |
| TIAF1 | -1.331460689 | 0.0001757 | -1.33110717 | 0.000754344 |
| C1QTNF6 | -1.335773447 | 1.94E-24 | -1.480127933 | 1.03E-22 |
| C7orf60 | -1.340852719 | 8.05E-17 | -1.163705033 | 2.12E-15 |
| ACOX2 | -1.348842294 | 4.94E-13 | -3.471278734 | 8.78E-12 |
| KCNN2 | -1.355264858 | 0.000494262 | -1.777228035 | 0.001920842 |
| NUDT17 | -1.367498924 | 7.01E-13 | -2.928709122 | 1.23E-11 |
| GYG1 | -1.372047088 | 3.33E-26 | -1.093023939 | 1.96E-24 |
| DSC2 | -1.373972792 | 5.29E-11 | -1.16564916 | 7.34E-10 |
| ZNF548 | -1.374605878 | 5.52E-10 | -1.129381172 | 6.70E-09 |
| RNF38 | -1.376713081 | 4.28E-16 | -1.175558021 | 1.05E-14 |
| NAP1L5 | -1.382097814 | 6.07E-15 | -1.613330546 | 1.33E-13 |
| GPNMB | -1.38320472 | 1.60E-10 | -1.819585384 | 2.10E-09 |
| YY2 | -1.393808513 | 9.70E-05 | -1.099972131 | 0.000448018 |
| SRPX2 | -1.395439338 | 5.93E-22 | -4.280805482 | 2.49E-20 |
| H2AFJ | -1.405989443 | 2.20E-16 | -1.745286055 | 5.50E-15 |
| ZBTB5 | -1.411531277 | 6.54E-27 | -1.623218383 | 4.11E-25 |
| GNB4 | -1.418157194 | 4.59E-09 | -1.933900625 | 4.82E-08 |
| KIAA1324 | -1.420110896 | 3.75E-08 | -2.408319118 | 3.41E-07 |
| CBX5 (HP1a) | -1.425154274 | 4.46E-12 | -1.456530174 | 7.00E-11 |
| FAM126B | -1.426850111 | 1.30E-15 | -1.079991173 | 3.04E-14 |
| TDG | -1.429889028 | 6.07E-29 | -1.949156213 | 4.30E-27 |
| HHLA3 | -1.430389233 | 1.95E-11 | -2.360142131 | 2.85E-10 |
| GPD2 | -1.448818457 | 7.55E-19 | -1.600506958 | 2.37E-17 |
| CLDN1 | -1.448981855 | 1.08E-29 | -1.122360161 | 8.15E-28 |
| ANG | -1.45099278 | 7.27E-16 | -1.354376872 | 1.74E-14 |
| VEZT | -1.45210288 | 5.58E-30 | -1.879948776 | 4.23E-28 |
| NCOA7 | -1.454651643 | 5.98E-22 | -1.542529384 | 2.50E-20 |
| TMCC3 | -1.455394889 | 4.94E-07 | -2.58871302 | 3.72E-06 |
| AMPD3 | -1.474670275 | 1.50E-17 | -3.259599596 | 4.27E-16 |
| GBP2 | -1.475665546 | 5.14E-25 | -2.625580688 | 2.80E-23 |
| PPT1 | -1.483342757 | 8.09E-32 | -0.954548595 | 6.92E-30 |
| SLC8A1 | -1.492179401 | 1.52E-10 | -1.061412963 | 2.00E-09 |
| PPP1R1C | -1.493931645 | 1.63E-12 | -2.046918213 | 2.74E-11 |
| C14orf182 | -1.49448622 | 1.49E-11 | -2.202179509 | 2.21E-10 |
| BCHE | -1.500480463 | 3.74E-24 | -3.895003579 | 1.91E-22 |
| PLAU | -1.502211042 | 1.10E-30 | -1.971506164 | 8.68E-29 |
| PIGV | -1.503618295 | 6.75E-26 | -1.378091434 | 3.93E-24 |
| RNF141 | -1.520193833 | 3.06E-23 | -2.001609941 | 1.43E-21 |
| BTD | -1.521221791 | 1.37E-19 | -1.421058333 | 4.72E-18 |
| SYTL5 | -1.525558884 | 7.24E-16 | -4.225052129 | 1.74E-14 |
| BEST3 | -1.527368281 | 1.96E-07 | -2.58455009 | 1.58E-06 |
| TGFB3 | -1.544386528 | 1.01E-06 | -1.316888762 | 7.18E-06 |
| S100A3 | -1.553157734 | 7.51E-17 | -1.119536941 | 1.98E-15 |
| HIST1H2BC | -1.561997782 | 4.16E-08 | -2.09178596 | 3.75E-07 |
| EDEM3 | -1.566065711 | 1.39E-16 | -1.200919634 | 3.57E-15 |
| CAB39L | -1.566866032 | 2.06E-14 | -2.236350974 | 4.28E-13 |
| DIXDC1 | -1.570622488 | 4.37E-17 | -1.007469213 | 1.18E-15 |
| GPM6A | -1.571783668 | 1.71E-06 | -2.992890938 | 1.15E-05 |
| HSD11B1 | -1.574808092 | 7.17E-05 | -1.534607714 | 0.000340306 |
| CREB5 | -1.58389974 | 6.18E-10 | -1.422270673 | 7.44E-09 |
| GPX3 | -1.585233123 | 1.15E-23 | -1.912986756 | 5.59E-22 |
| ZBTB41 | -1.589947537 | 3.27E-12 | -1.714781198 | 5.29E-11 |
| SERPINA1 | -1.59446725 | 2.24E-11 | -1.889958953 | 3.27E-10 |
| AASDHPPT | -1.597311848 | 1.06E-31 | -1.576520513 | 9.05E-30 |
| SAMD13 | -1.597588952 | 1.29E-09 | -2.88270572 | 1.48E-08 |
| CCDC117 | -1.600595259 | 5.06E-32 | -1.500571801 | 4.36E-30 |
| SGMS2 | -1.607393522 | 4.23E-15 | -1.000220466 | 9.44E-14 |
| PTK2 | -1.613197445 | 5.19E-33 | -1.369902381 | 4.76E-31 |
| ACBD5 | -1.617161954 | 1.25E-26 | -1.400319251 | 7.71E-25 |
| CPNE8 | -1.624986997 | 2.96E-24 | -2.125747351 | 1.52E-22 |
| CATSPER1 | -1.633672736 | 7.87E-05 | -2.590003138 | 0.000370955 |
| TBC1D13 | -1.6445179 | 3.20E-32 | -1.467131128 | 2.80E-30 |
| TMEM38B | -1.647960315 | 4.46E-24 | -1.40541051 | 2.26E-22 |
| ARSD | -1.655630689 | 1.05E-37 | -1.992044666 | 1.30E-35 |
| VWA7 | -1.662047965 | 1.75E-15 | -2.248623682 | 4.03E-14 |
| RBMS1 | -1.668205141 | 1.16E-40 | -1.747036755 | 1.70E-38 |
| MARCKS | -1.679605898 | 3.88E-42 | -2.46363287 | 6.10E-40 |
| FERMT2 | -1.681727654 | 1.83E-32 | -1.424638284 | 1.61E-30 |
| CCDC43 | -1.687364359 | 1.73E-39 | -1.828701708 | 2.39E-37 |
| KRT15 | -1.68754815 | 3.91E-07 | -3.931153853 | 2.99E-06 |
| CXorf57 | -1.699054649 | 3.13E-12 | -1.278993824 | 5.08E-11 |
| HMGN5 | -1.707938599 | 4.56E-10 | -1.13228867 | 5.59E-09 |
| ETNK1 | -1.711773637 | 4.56E-26 | -1.636294956 | 2.66E-24 |
| NR0B1 | -1.722499145 | 3.32E-17 | -3.694850371 | 9.13E-16 |
| FBXO45 | -1.746095051 | 2.00E-26 | -2.214437481 | 1.21E-24 |
| HECTD3 | -1.753173521 | 9.54E-41 | -1.398532784 | 1.41E-38 |
| DPYD | -1.755668506 | 2.73E-25 | -1.504495481 | 1.51E-23 |
| NAP1L2 | -1.759255532 | 5.70E-24 | -2.550468207 | 2.87E-22 |
| G0S2 | -1.765939797 | 4.02E-20 | -0.937268041 | 1.45E-18 |
| ASL | -1.767140706 | 7.46E-36 | -1.504760974 | 8.13E-34 |
| ATL3 | -1.770210841 | 9.17E-42 | -1.665742801 | 1.39E-39 |
| AKR1B10 | -1.775419119 | 9.84E-37 | -2.726468443 | 1.18E-34 |
| SYTL2 | -1.791304631 | 2.05E-38 | -1.952596997 | 2.64E-36 |
| TTC39A | -1.794513454 | 5.77E-22 | -1.848923703 | 2.43E-20 |
| TLR1 | -1.794972763 | 5.57E-15 | -4.51130201 | 1.23E-13 |
| C19orf82 | -1.800839895 | 2.86E-19 | -2.097380014 | 9.55E-18 |
| ANXA9 | -1.808571015 | 1.24E-12 | -1.893751548 | 2.10E-11 |
| GXYLT1 | -1.80972745 | 2.99E-21 | -1.122125543 | 1.17E-19 |
| DYNLT3 | -1.821698003 | 1.37E-39 | -1.380537497 | 1.92E-37 |
| WSB2 | -1.82358111 | 5.81E-46 | -1.863924312 | 1.09E-43 |
| ACER2 | -1.827968458 | 5.11E-08 | -1.192446516 | 4.52E-07 |
| LOXL4 | -1.837745515 | 3.94E-22 | -1.904149538 | 1.68E-20 |
| RMND5A | -1.837837583 | 1.05E-30 | -1.378016071 | 8.37E-29 |
| COQ5 | -1.846572011 | 1.30E-36 | -3.146203094 | 1.53E-34 |
| FNDC3A | -1.859323393 | 6.53E-22 | -1.613143139 | 2.71E-20 |
| CFL2 | -1.882072902 | 8.25E-49 | -2.014887122 | 1.70E-46 |
| INSIG1 | -1.885109607 | 8.21E-51 | -1.090891824 | 1.87E-48 |
| EFNA1 | -1.888359679 | 1.39E-30 | -1.930028562 | 1.09E-28 |
| FRMD3 | -1.893854988 | 2.53E-11 | -1.471835575 | 3.64E-10 |
| STC1 | -1.915788055 | 7.47E-43 | -5.418633937 | 1.21E-40 |
| IKZF2 | -1.933197502 | 1.62E-10 | -1.38077695 | 2.12E-09 |
| C15orf48 | -1.986291358 | 2.22E-34 | -1.99092788 | 2.27E-32 |
| OTUD6B | -2.005784691 | 1.21E-29 | -2.354599675 | 9.00E-28 |
| FNIP2 | -2.026258391 | 8.73E-20 | -1.588530751 | 3.06E-18 |
| CLEC2B | -2.057490571 | 2.34E-17 | -4.72528995 | 6.52E-16 |
| FRK | -2.058585183 | 9.26E-27 | -1.831745857 | 5.79E-25 |
| AKAP6 | -2.090352576 | 2.11E-20 | -1.567671361 | 7.74E-19 |
| TCN2 | -2.093014308 | 1.61E-36 | -2.726313455 | 1.87E-34 |
| ZNF35 | -2.11083676 | 1.28E-19 | -1.15713394 | 4.40E-18 |
| CACNA2D1 | -2.121806639 | 8.05E-25 | -1.772467454 | 4.36E-23 |
| SEMA3E | -2.150390526 | 3.62E-23 | -2.165060892 | 1.67E-21 |
| CADM2 | -2.155540265 | 9.33E-16 | -2.601390369 | 2.21E-14 |
| NCALD | -2.21308328 | 1.21E-31 | -1.394694323 | 1.02E-29 |
| TMEM140 | -2.289436372 | 1.32E-37 | -1.771865323 | 1.62E-35 |
| PPAP2B | -2.305690358 | 2.29E-57 | -3.213272534 | 6.63E-55 |
| LYPLA1 | -2.313785942 | 9.77E-56 | -2.589943724 | 2.71E-53 |
| SHH | -2.438234535 | 1.60E-27 | -1.699994762 | 1.04E-25 |
| HSPB8 | -2.442399227 | 1.52E-67 | -1.568951949 | 6.68E-65 |
| SORL1 | -2.488344064 | 2.62E-24 | -1.022210948 | 1.36E-22 |
| NR1H4 | -2.491640863 | 4.98E-36 | -2.037822567 | 5.57E-34 |
| SOX6 | -2.527457955 | 2.82E-22 | -1.115845486 | 1.21E-20 |
| TSPAN8 oncogenic | -2.5423795 | 5.41E-39 | -2.948453787 | 7.34E-37 |
| NCR3LG1 | -2.554889348 | 8.31E-12 | -1.518299423 | 1.27E-10 |
| IL1RAP | -2.569342116 | 4.34E-35 | -2.052484372 | 4.58E-33 |
| CPM | -2.648935569 | 3.34E-51 | -1.828456944 | 7.75E-49 |
| PAG1 | -2.674379829 | 3.84E-21 | -0.945340222 | 1.49E-19 |
| TMEM117 | -2.856236626 | 2.29E-29 | -5.141427001 | 1.66E-27 |
| EHMT2 | -2.92502166 | 1.02E-64 | -3.802484955 | 3.95E-62 |
| **PBLD** | **-2.950581438** | **7.95E-61** | **-2.870229157** | **2.53E-58** |
| SNTB1 | -3.140690157 | 2.60E-63 | -1.455584926 | 9.47E-61 |
| VEPH1 | -3.325005823 | 6.52E-31 | -2.502983086 | 5.22E-29 |
| DPP4 | -3.461393426 | 1.89E-52 | -2.817855895 | 4.54E-50 |
| PTX3 | -4.562368942 | 3.74E-107 | -1.14105843 | 4.76E-104 |

*The mRNA expression levels of genes in G9a-attenuated lung cancer cells are presented as log2 ratio to that of lung cancer cells transfected with control siRNA. If the absolute log2 ratio of both cells were no less or more than 1.0, the gene would be listed.
