# Supplementary material for: Targeting histone methyltransferase G9a inhibits growth and Wnt signaling pathway by epigenetically regulating HP1α and APC2 gene expression in non-small cell lung cancer
Source: Mol Cancer. 2018 Oct 22;17:153. doi: 10.1186/s12943-018-0896-8 (PMC6198520; doi:10.1186/s12943-018-0896-8)
Supplement: Supplementary file 2 — Figure S1. Inhibition of G9a induces cell cycle arrest. a A549 in G1 phase b H1299 in G2 phase. The statistical analyses of G1 and sub-G1 are shown (* P < 0.05 compared to control siRNA). Figure S2. Impact of knockdown of G9a on the levels of p53 and c-Myc proteins in A549 (p53 wild-type), H1299 (p53 null), and H1975 (p53 mutated) cancer cells. Figure S3. Impact of knockdown of HP1α on NSCLC cells proliferation. a Western blot of HP1α in three cell lines. b Cell proliferation after 72 h posttransfection of HP1α siRNA. (*P < 0.05, **P < 0.01, compared to cells transfected with control siRNA. Figure S4. Correlation between the level of G9a mRNA with that of DKK1 mRNA and WIF1 mRNA. The statistical analysis showed that the level of G9a mRNA level is reversely correlated with that of a DKK1 mRNA (P = 0.00828, R = - 0.38), b WIF1 mRNA (P = 0.0403, R = -0.3) in 46 of LUSC tissues. X-axis is for the normalized G9a (EMHT2) mRNA level, Y-axis is for the normalized DKK1/WIF1 mRNA level. Figure S5. Unsupervised hierarchical clustering analysis of selected upregulated genes upon G9a knockdown. These genes include ICAM5, JPH3, MGMT, MMP2, RASSF1α etc. that are frequently silenced by epigenetic mechanisms in lung cancers. The two top rows represent two independent siRNA repeats, Ctrl1/2 is for control siRNA and G9a-KD1/2 is for G9A siRNA1/2. Figure S6. UNC0638 treatment decreased HP1α in H1299 cells. Cells were treated with defined UNC0638 for 72 hours and then protein was extracted for Western blot analysis of HP1 α, G9a, H3K9-Me2. (PDF 438 kb) [file 12943_2018_896_MOESM2_ESM.pdf]

Figure S1

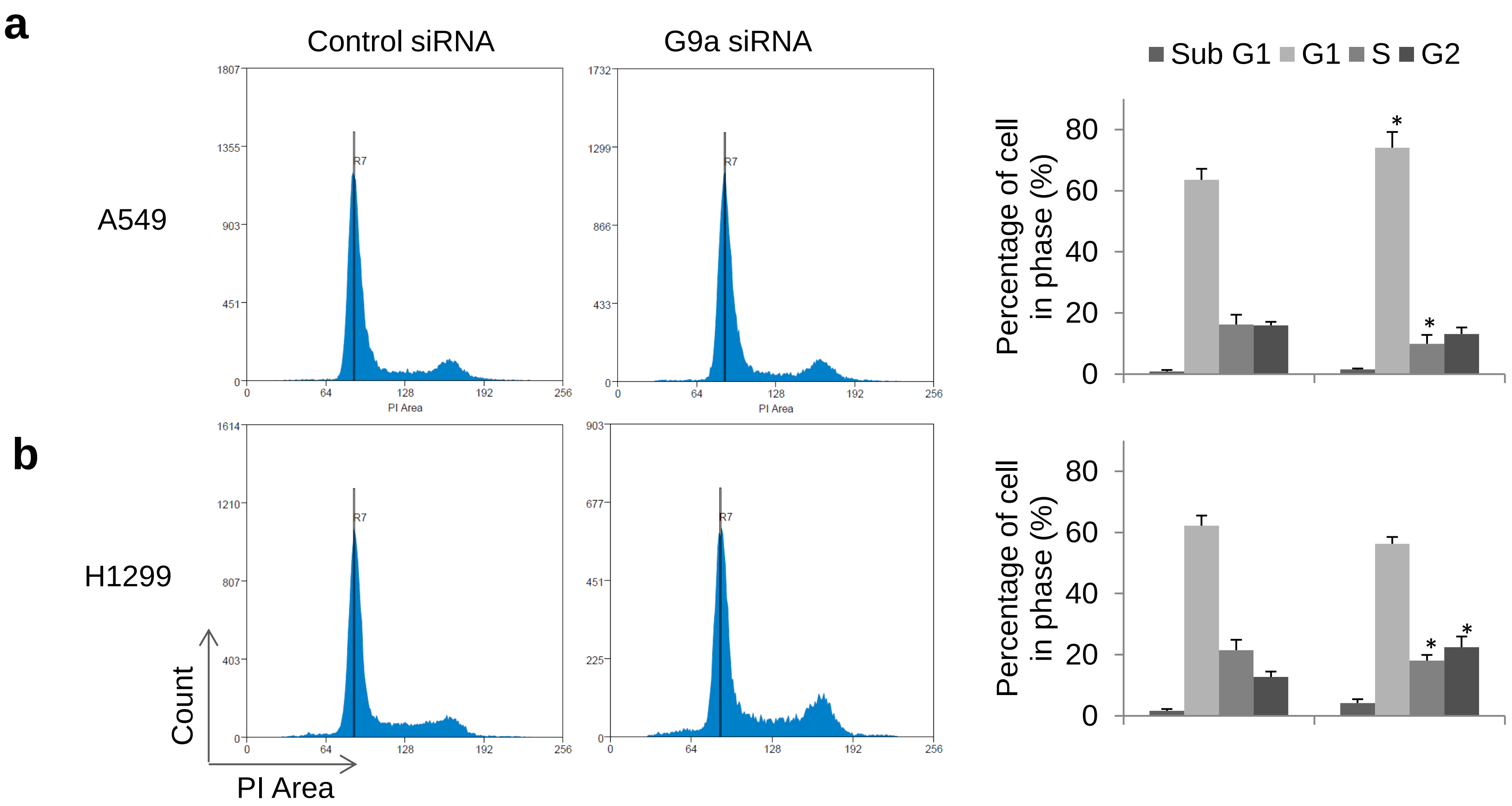

Figure S1. **Inhibition of G9a induces cell cycle arrest. a** A549 in G1 phase **b** H1299 in G2 phase. The statistical analyses of G1 and sub-G_1 are shown (* P < 0.05 compared to control siRNA).

Figure S2

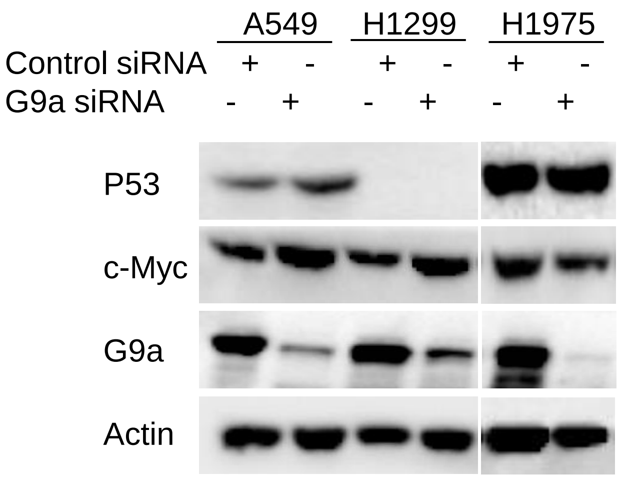

Figure S2. **Impact of knockdown of G9a on the levels of p53 and c-Myc proteins in A549** (p53 wild-type), H1299 (p53 null), **and H1975** (p53 mutated) **cancer cells.**

Figure S3

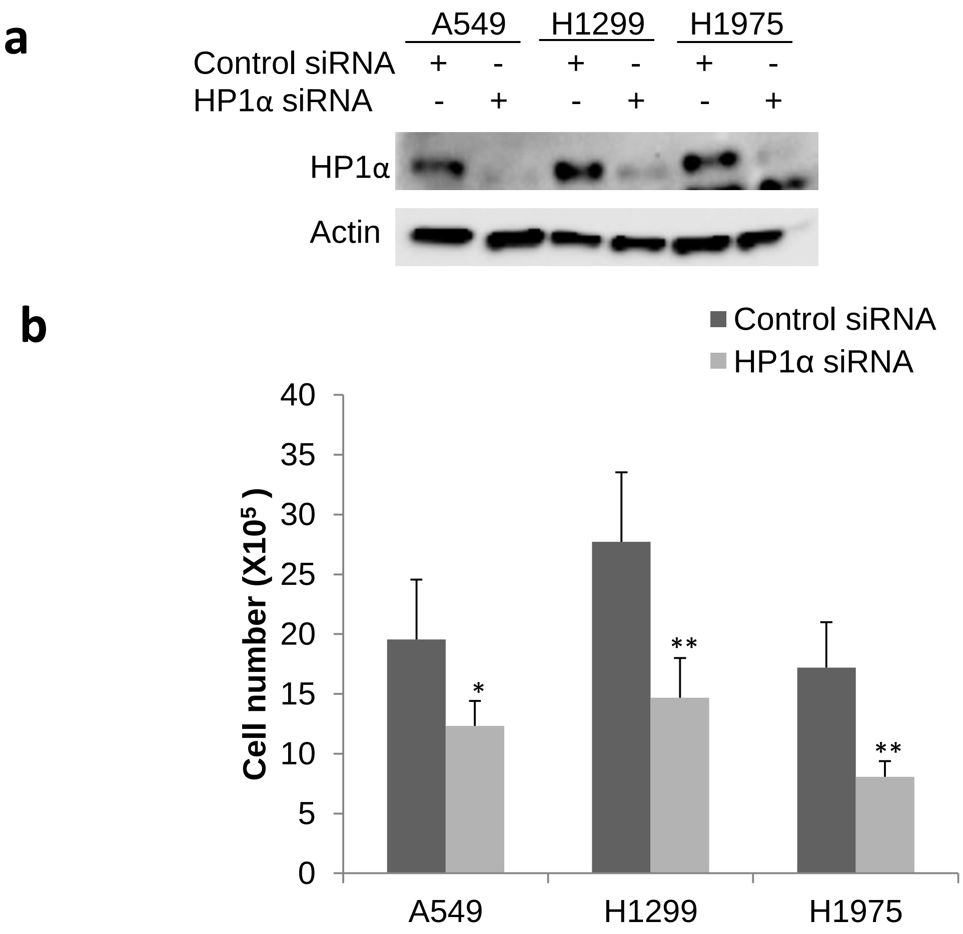

Figure S3. **Impact of knockdown of HP1α on NSCLC cells proliferation. a** Western blot of HP1α in three cell lines. **b** Cell proliferation after 72 hours posttransfection of HP1α siRNA. (* P<0.05, **P <0.01, compared to cells transfected with control siRNA.

Figure S4

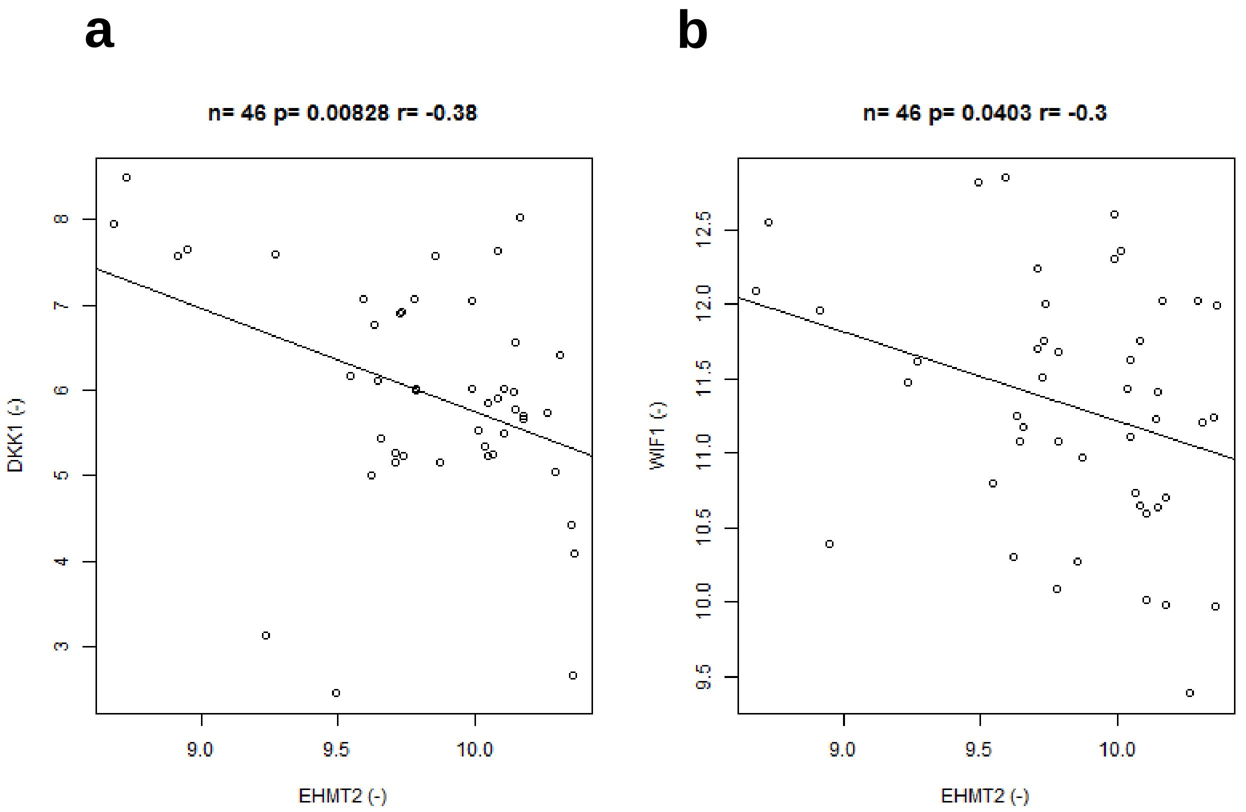

Figure S4. Correlation between the level of G9a mRNA with that of DKK1 mRNA and WIF1 mRNA. The statistical analysis showed that the level of G9a mRNA level is reversely correlated with that of **a** DKK1 mRNA (*P* = 0.00828, R = - 0.38), **b** WIF1 mRNA (*P* = 0.0403, R = -0.3) in 46 of LUSC tissues. X-axis is for the normalized G9a (EMHT2) mRNA level, Y-axis is for the normalized DKK1/WIF1 mRNA level.

Figure S5

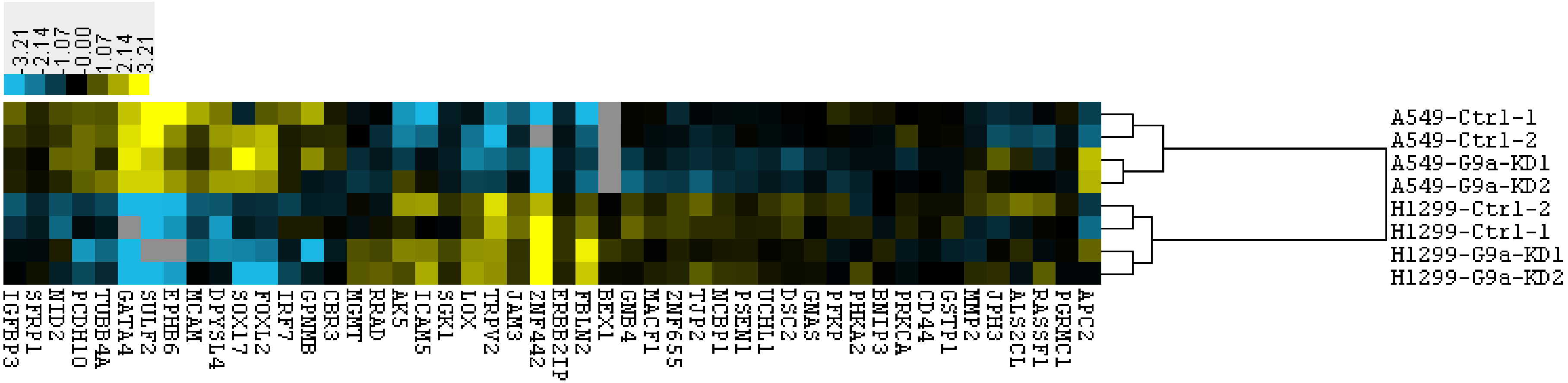

Figure S5. Unsupervised hierarchical clustering analysis of selected upregulated genes upon G9a knockdown. These genes include ICAM5, JPH3, MGMT, MMP2, RASSF1α etc. that are frequently silenced by epigenetic mechanisms in lung cancers. The two top rows represent two independent siRNA repeats, Ctrl1/2 is for control siRNA and G9a-KD1/2 is for G9A siRNA1/2.

Figure S6

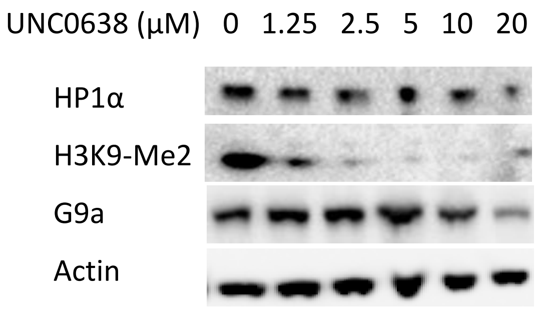

Figure S6. **UNC0638 treatment decreased HP1α in H1299 cells.** Cells were treated with defined UNC0638 for 72 hours and then protein was extracted for Western blot analysis of HP1 α, G9a, H3K9-Me2
